# Exploration of the Potential Bioactive Compounds and Functional Mechanism of Chaihu Sanshen Capsule in Ameliorating Myocardial Ischaemia–Reperfusion Injury: A Serum Pharmaco‐Chemistry With Network Pharmacology Analysis

**DOI:** 10.1111/jcmm.70666

**Published:** 2025-07-28

**Authors:** Weisong Wang, Zengyu Zhang, Rongzhen Liu, Yu Zheng, Yaqi Hu, Xia Li, Zihao Shen, Hengyou Yuan, Jianhe Liu

**Affiliations:** ^1^ Department of Cardiology The First Hospital of Hunan University of Chinese Medicine Changsha Hunan China; ^2^ Research Center for Clinical Medicine Jinshan Hospital Affiliated to Fudan University Shanghai China; ^3^ Literature and Information Research Institute Hunan Academy of Chinese Medicine Changsha Hunan China; ^4^ Department of Respiratory Diseases, Medical School Hunan University of Chinese Medicine Changsha Hunan China; ^5^ Laboratory of Vascular Biology and Translational Medicine, Medical School Hunan University of Chinese Medicine Changsha Hunan China; ^6^ Xiangxing College Hunan University of Chinese Medicine Changsha Hunan China

**Keywords:** Chaihu Sanshen capsule, myocardial ischaemia–reperfusion injury, network pharmacology, PI3k/Akt/p53 signalling pathway, serum pharmaco‐chemistry

## Abstract

Previous studies have shown the potential of Chaihu Sanshen capsule (CHSSC) to ameliorate myocardial ischemia‐reperfusion injury (MIRI), but there is yet no corresponding research on its chemical ingredients and multi‐target action network. The study aims to identify the chemical composition and potential bioactive compounds of CHSSC and elucidate its underlying mechanisms in MIRI treatment. Ultra‐high‐performance liquid chromatography‐Q exactive focus‐mass spectrometry was used to analyse the chemical composition and potential bioactive compounds of CHSSC. The active compounds were analysed via network pharmacology to identify the core targets and pathways. The oxygen–glucose deprivation/reoxygenation (OGD/R) H9C2 cell model and MIRI rat model were established, followed by intervention with CHSSC. TUNEL, flow cytometry and western blotting assays were used to observe the effects of CHSSC on apoptosis, pyroptosis and the PI3K/AKT/p53 signalling pathway, respectively, of cardiomyocytes. In all, 1587 compounds were detected in CHSSC, of which 106 were absorbed into the bloodstream, mainly comprising flavonoids, terpenoids, alkaloids, organic acids, coumarins and phenols. CHSSC primarily targeted TP53, AKT, STAT3, HSP90AA1 and MAPK and involved the regulation of p53, PI3K/AKT, JAK2/STAT3 and MAPK signalling pathways; however, these predicted targets have not yet been validated by confirmatory binding assays. In vitro experiments showed that CHSSC reduced the apoptosis and pyroptosis rates of OGD/R H9C2 cells. In vivo, CHSSC ameliorated myocardial injury in MIRI rats, decreased the cardiomyocyte apoptosis rate, increased PI3K and AKT phosphorylation and inhibited p53 phosphorylation. In conclusion, this study elucidated the potential bioactive compounds and multi‐targets action network of CHSSC in mitigating MIRI, and verified that the effects of CHSSC on MIRI are link to the PI3K/AKT/p53 signalling pathway.

**Abbreviations**
BC: betweenness centrality
BP: biological process
CC: cellular component
CC: closeness centrality
CHSSC: Chaihu Sanshen capsule
CK‐MB: creatine kinase‐MB isoenzymes
cTnI: troponin I
DC: degree centrality
DL: drug‐likeness
DSF: disulfiram
ECG: electrocardiograph
GSDMD: gasdermin D
HE: haematoxylin and eosin
LDH: lactate dehydrogenase
MF: molecular function
MIRI: myocardial ischaemia–reperfusion injury
NC: neighbourhood connectivity
OB: oral bioavailability
OGD/R: oxygen–glucose deprivation/reoxygenation
ROS: reactive oxygen species
TCM: traditional Chinese medicine
TCMSP: traditional Chinese medicine system and the analysis platform
TUNEL: terminal deoxynucleotidyl transferase‐mediated nick end labelling
UHPLC‐QE‐MS: ultra‐high‐performance liquid chromatography‐Q exactive focus‐mass spectrometry

## 1 Introduction

According to a report from the World Health Organization, ischaemic heart disease remains the leading cause of death worldwide, accounting for 16% of all deaths. Since 2000, the most significant increase in mortality has been attributed to ischaemic heart disease, with deaths rising by over 2 million in 2019, reaching a total of 8.9 million [1]. Timely and effective reperfusion therapy can swiftly restore blood flow to the ischaemic myocardium, preserve viable myocardial cells, reduce infarction size and enhance patient survival [2]. However, myocardial ischaemia–reperfusion injury (MIRI) may ensue upon restoration of blood flow, resulting in additional or potentially fatal damage to the heart, by means of severe arrhythmias, increased infarction size and deteriorated ventricular function, all of which notably reduced the clinical efficacy and prognosis of reperfusion therapy [3]. Therefore, it is imperative to prevent and mitigate MIRI by identifying effective targets and medications to enhance clinical outcomes and reduce mortality rates associated with ischaemic heart disease.

Multiple mechanisms contribute to MIRI, including mitochondrial dysfunction, metabolic alterations, inflammation, programmed cell death, excessive reactive oxygen species (ROS) production and autophagy imbalance and interventions targeting these different mechanisms can alleviate reperfusion injury [4, 5, 6]. Despite recent studies demonstrating promising advancements, there remains an urgent clinical need to develop effective strategies for the prevention and treatment of MIRI. Studies have shown that various Chinese herbal compounds can reduce MIRI [7]. For instance, ginseng and its bioactive compounds can regulate proteins associated with oxidative stress, inflammatory cytokines and apoptotic factors, thereby protecting the myocardium and relieving MIRI [8], HJ11 decoction controlled the development of MIRI by regulating ACSL4‐mediated ferroptosis [9], and Huang Qi Tong Bi decoction reduce the levels of circulating proinflammatory cytokines by the regulating the HMGB1/TLR/NF‐κB signalling pathway [10]. However, studies on Chinese herbal compounds face challenges such as the uncertainty of effective ingredients or active compounds, the lack of specific target points and insufficient interconnections with multiple target points. The application of serum pharmaco‐chemistry and network pharmacology in its research can effectively address these issues. Integrating pharmaco‐chemistry with network pharmacology analysis enables the identification of effective blood‐absorbing compounds of Chinese herbs, along with their associated target points and the interaction network comprising ‘drug‐active compound‐target point‐disease’ [11]. This approach holds promise for uncovering more effective clinical treatment targets and discovering related active compounds.

Chaihu Sanshen capsule (CHSSC) contains *Bupleuri Radix*, *Scutellariae Radix*, *Salviae Miltiorrhizae Radix Et Rhizoma* among other compounds (full list provided in Table 1, the plant name has been checked with http://www.theplantlist.org) and is a compound preparation of Chinese herbs formulated under the guidance of traditional Chinese medicine (TCM) theory. It has the effect of moving qi, activating blood and resolving phlegm. Studies have shown that *Salviae Miltiorrhizae Radix Et Rhizoma* and its components offer protection against a range of cardiovascular diseases including myocardial infarction, MIRI, arrhythmia, cardiac hypertrophy and myocardial fibrosis [12]. Scutellarin, a bioactive compound of *Scutellariae Radix*, has been shown to attenuate MIRI by reducing cellular apoptosis and oxidative stress [13]. Our previous studies have shown that CHSSC can mitigate intracellular calcium overload and ameliorate ventricular arrhythmias following myocardial ischaemia–reperfusion by inhibiting the CaMKII/FKBP12.6/RyR2 signalling pathway [14]. Additionally, it can attenuate oxidative stress and inhibit myocardial cell apoptosis to confer a protective effect against MIRI [15]. However, the chemical composition and bioactive compounds of CHSSC remain unknown. Further exploration is therefore necessary to identify these compounds, their target points and their interaction network based on our previous studies.

**TABLE 1:** Chinese herbs of CHSSC.

Chinese name	English name	Latin name	Drug proportion
Chai Hu	Bupleuri Radix	*Bupleurum chinense* DC.	15
Dan Shen	Salviae Miltiorrhizae Radix Et Rhizoma	*Salvia miltiorrhiza* Bge.	10
Fa Ban Xia	Pinelliae Rhizoma Preparatum	*Pinellia ternata* (Thunb.) Makino	10
Ku Shen	Sophorae Flavescentis Radix	*Sophora flavescens* Ait.	10
Qing Hao	Artemisiae Annuae Herba	*Artemisia annua* L.	10
Dang Shen	Codonopsis Radix	*Codonopsis pilosula* (Franch.) Nannf.	15
Gan Cao	Glycyrrhiza Radix Et Rhizoma	*Glycyrrhiza uralensis* Fisch.	6
Huang Qin	Scutellariae Radix	*Scutellaria baicalensis* Georgi	6

In this study, we initially employed ultra‐high‐performance liquid chromatography‐Q exactive focus‐mass spectrometry (UHPLC‐QE‐MS) for the analysis and identification of the chemical constituents in CHSSC. Subsequently, serum pharmaco‐chemistry analysis was used to evaluate the potential active components of CHSSC that are absorbed into the serum, elucidate the material basis of their effects and serve as a means of quality control. Following this, network pharmacology analysis was conducted using the identified active compounds to determine their targets and construct a multi‐level network encompassing ‘Chinese herbal compound preparation‐active compound‐target points‐pathway‐diseases’. Finally, we established a rat model of MIRI to further validate the core action pathway and elucidate the functional mechanism of CHSSC in the treatment of MIRI.

## 2 Materials and Methods

### 2.1 Composition and Preparation of CHSSC

CHSSC was provided by the Pharmaceutical Preparation Room of the First Hospital of Hunan University of Chinese Medicine (lot#: 20230719) and the clinical dosage of it is established at 4.5 g/day (1.5 g orally, three times daily) for adults in clinical practice. The specific components of CHSSC are shown in Table 1. The capsule's contents were dissolved in distilled water for use, and a medium dose of CHSSC (0.43 g/kg/day) was administered corresponding to the clinical dose that was calculated by conversion, based on the human‐to‐rat body surface area ratio (human body weight was set as 60 kg).

### 2.2 Reagents

Serum lactate dehydrogenase (LDH) assay kit (#A020‐2‐2) was bought from Nanjing Jiancheng Bioengineering Institute (Nanjing, China). Serum troponin I (cTnI) and serum creatine kinase‐MB isoenzymes (CK‐MB) ELISA kits (#CSB‐E08594r, #CSB‐E14403r) were from Wuhan Huamei Biological Engineering Co. Ltd. (Wuhan, China). TUNEL assay kits (#KGA704, #40306ES50) were purchased from Jiangsu Kaiji Biotechnology Co. Ltd. and Yeasen Biotechnology Co. Ltd. (Shanghai, China). Endogenous avidin‐biotin blocking kit (#IH0125) was from Beijing Leagene Biotechnology Co. Ltd. Cell Counting Kit‐8 (#NU679) was from DOJINDO Laboratories (Kumamoto, Japan). Z‐VAD‐FMK (#HY‐16658B) and DSF (#HY‐B0240) were from MedChemExpress LLC (Shanghai, China). Anti‐AKT (#AWA10126), anti‐p‐AKT (#AWA45006), anti‐p53 (#AWA42607), anti‐p‐p53 (#AWA42602), anti‐PI3K (#AWA11331), anti‐p‐PI3K (#AWA41249), HRP goat anti‐mouse IgG (H + L) secondary antibody (#AWS0001) and HRP goat anti‐rabbit IgG (H + L) secondary antibody (#AWS0002) were from Changsha Abiowell Biotechnology Co. Ltd. (Changsha, China). Anti‐Bax (#50599‐2‐Ig), anti‐caspase 3 (#66470‐2‐Ig), anti‐caspase 1 (#22915‐1‐AP), anti‐caspase 7 (#27155‐1‐AP), anti‐caspase 8 (#13423‐1‐AP), anti‐IL‐18 (10663‐1‐AP), anti‐β‐actin (#66009‐1‐Ig) and anti‐GAPDH (#10494‐1‐AP) were from Proteintech (Chicago, USA). Anti‐IL‐1β was from Abcam (Cambridge, UK).

### 2.3 H9C2 Cells

H9C2 cells were purchased from Changsha Abiowell Biotechnology Co. Ltd. (Changsha, China). The H9C2 cells were cultured in DMEM supplemented with 10% FBS and 1% antibiotic (penicillin/streptomycin) at 37°C, 5% CO_2_ and under conditions of saturated humidity in an incubator.

### 2.4 Animals

Sixty 8‐week‐old SD male rats (body weight: 200 ± 20 g) were purchased from Hunan Silaike Jingda Animal Co. Ltd. (certification No.: SCXK [Xiang] 2019‐0004). All rats were raised under specific pathogen‐free conditions at temperature of 25°C±1°C and humidity of 50%±5%, with a 12‐h light/dark cycle (certification No.: SCXK [Xiang] 2020‐0010). All animal experiments were conducted in accordance with the National Institutes of Health (NIH) Guide for the Care and Use of Laboratory Animals and were approved by the Ethics Committee for Experimental Animals of the First Hospital of Hunan University of Chinese Medicine.

### 2.5 CHSSC Chemical Ingredients and Its Serum Pharmaco‐Chemistry Analysis

#### 2.5.1 Preparation of CHSSC Samples

Three CHSSC capsules were randomly selected from the same batch of capsules. Briefly, 100 mg of the CHSSC samples were added to 500 μL of extracted solution dissolved in 80% methanol containing 10 μg/mL of internal standard. After 30‐s vortex, the samples were homogenised at 45 Hz for 4 min and sonicated for 1 h in an ice‐water bath. After placing for 1 h in −40°C, the samples were centrifuged at 13,800 × *g* for 15 min at 4°C. The supernatant was carefully filtered through a 0.22‐μm microporous membrane; 100 μL from each sample was taken and pooled as the QC sample. The samples were stored at −80°C until UHPLC–MS analysis.

#### 2.5.2 Preparation of Rat Serum Samples

Six rats (200 ± 20 g) were randomly divided into two groups: CHSSC‐treated serum group (*n* = 3) and blank serum group (*n* = 3). The CHSSC group was orally administered CHSSC at a dosage of 0.86 g/kg/day, and the blank group was given the same amount of distilled water for seven consecutive days. Blood samples were collected from the rat abdominal aorta after 2 h of the last administration, and then centrifuged to obtain rat serum samples.

Briefly, 400 μL serum sample was added to 40 μL hydrochloric acid (2 mol/L), and the mixture was vortexed for 1 min, followed by incubation for 15 min at 4°C. The vortex and incubation cycle were repeated four times. Then, 1.6 mL acetonitrile was added, and the samples were centrifuged at 13,800 × *g* for 5 min at 4°C. Next, 1800 μL of this supernatant was transferred to a fresh tube and nitrogen dried. The dried samples were reconstituted in 150 μL 80% methyl alcohol containing 10 μg/mL of the internal standard by vortexing for 5 min. The constitution was then re‐centrifuged at 13,800 × *g* for 5 min at 4°C, and 120 μL of the supernatant was transferred to a fresh glass vial for LC/MS analysis.

#### 2.5.3 UHPLC‐QE‐MS Analysis Conditions

LC–MS/MS analysis was performed on an UHPLC system (Vanquish, Thermo Fisher Scientific) with a Waters UPLC BEH C18 column (1.7 μm, 2.1 × 100 mm) (Waters, Massachusetts, USA). Specifically, the flow rate was set at 0.4 mL/min, and the sample injection volume was set at 5 μL. The mobile phase consisted of 0.1% formic acid in water (A) and 0.1% formic acid in acetonitrile (B). The multi‐step linear elution gradient programme was as follows: 0–3.5 min, 95%–85% A; 3.5–6 min, 85%–70% A; 6–6.5 min, 70%–70% A; 6.5–12 min, 70%–30% A; 12–12.5 min, 30%–30% A; 12.5–18 min, 30%–0% A; 18–25 min, 0%–0% A; 25–26 min, 0%–95% A; 26–30 min, 95%–95% A.

An Q Exactive Focus‐mass spectrometer coupled with Xcalibur software (Thermo Fisher Scientific, Massachusetts, USA) was employed to obtain the MS and MS/MS data based on the IDA acquisition mode. During each acquisition cycle, the mass range was from 100 to 1500. Sheath gas flow rate: 30 Arb, Aux gas flow rate: 10 Arb, capillary temperature: 350°C, full MS resolution: 70,000, MS/MS resolution: 17,500, collision energy: 15/30/45 in NCE mode, spray voltage: 5.5 kV (positive) or −4.0 kV (negative).

#### 2.5.4 Identification of Compounds

The MS raw data were imported using XCMS software. Retention time correction, peak recognition, peak extraction, peak integration and peak alignment were carried out, and a self‐built secondary mass spectrometry database and corresponding fragmentation pattern matching method to identify substances in peaks containing MS/MS data were used.

#### 2.5.5 Venn Diagram Analysis of Serum Pharmaceutical Chemical Components

R software was used to draw the Venn diagram, which was used to display the metabolites of CHSSC, CHSSC‐treated serum and blank serum. Each ellipse in the Venn diagram represents a group and is mapped with a different colour, and the metabolite number of the intersection and non‐intersection are statistically displayed in the diagram. Venn diagrams are often used to identify metabolites that are common among multiple groups, which can visually represent the general state of medicinal ingredients absorbed into the blood.

#### 2.5.6 Correlation Analysis of Serum Pharmaceutical Chemical Components

We calculated the Pearson correlation coefficient and *p* value between two compounds using R software to measure their correlation degree. The degree of correlation between the two compounds is represented by the correlation coefficient ‘*r*’ which falls between −1 and 1. When positively correlated, the *r* value is between 0 and 1, and when negatively correlated, the *r* value is between −1 and 0. The absolute value of *r* closer to 1 represents a stronger correlation between two compounds; conversely, the absolute value of *r* closer to 0 shows weaker correlation.

### 2.6 Network Pharmacology Study

#### 2.6.1 Target Prediction

The bioactive compounds of CHSSC identified through serum pharmaco‐chemistry analysis were imported into the PubChem database (https://pubchem.ncbi.nlm.nih.gov/). The compound structure was downloaded, unified and standardised to its chemical name (Molecular Name). The oral bioavailability (OB) and drug‐likeness (DL) of these components were searched in the TCMSP database (https://www.tcmsp‐e.com/tcmsp.php) and compounds with OB (%) ≥ 30% and DL (%) ≥ 0.18% were selected for further analysis. Corresponding targets of these selected compounds were searched in the TCMSP database and were imported to Uniport database to search the corresponding gene symbol (search species was ‘HUMAN’) for subsequent study. Additionally, the Swiss Target Prediction database (http://www.swisstar‐getprediction.ch/) and Similarity ensemble approach database (https://sea.bkslab.org/) were used to predict the potential target of bioactive ingredients of CHSSC. MIRI‐related targets were obtained by searching (key word: ‘myocardial reperfusion injury’) and integrating the targets in five databases, including CTD (https://ctdbase.org/), Genecards (https://www.genecards.org/), DisGeNET (https://www.disgenet.org/), OMIM (https://omim.org/) and TTD (http://db.idrblab.net/ttd/).

#### 2.6.2 Construction and Analysis of Targets' Protein–Protein Interaction (PPI) Network

The intersection targets of drugs and diseases were imported into the String database (https://cn.string‐db.org/), and the species ‘ *Homo sapiens* ’ was selected. The PPI network was analysed in the String database by setting the minimum required interaction score to ‘highest confidence ≥ 0.900’, and hiding disconnected nodes in the network. Then the TSV file obtained from the PPI interaction analysis was imported into Cytoscape 3.9.1 software to present the normative PPI network diagram. Meanwhile, the Network Analyzer plug‐in was used to calculate the parameters, including degree centrality (DC), betweenness centrality (BC), closeness centrality (CC) and neighbourhood connectivity (NC), and set its median as a threshold to screen the core targets and construct the core target PPI network of the target hub.

#### 2.6.3 Enrichment Analysis of Gene Ontology (GO) and Kyoto Encyclopedia of Genes and Genomes (KEGG)

Analysed core targets were imported to the Metascape database (https://metascape.org/gp/index.html#/main/step1) for GO functional enrichment analysis (*p* < 0.01), and items of biological process (BP), cellular component (CC) and molecular function (MF) were obtained. The top 10 were presented by histograms and bubble charts. Core targets were also imported to the Metascape database for KEGG pathway enrichment analysis. Then, the results of the Metascape database were imported to the KEGG database for secondary classification of pathways.

#### 2.6.4 Molecular Docking Analysis

The entry IDs of the core targets ranked by degree value were retrieved from the UniProt database (https://www.uniprot.org/) and imported into the RCSB protein database (https://www.rcsb.org/) to search and download the pdb format file of the three‐dimensional structure of protein macromolecules. PyMol 2.5 software was used to pretreat the protein receptor structure and remove metal ions, water molecules and repeated chains in the protein structure. Then AutoDock 4.2.6 software was used to add hydrogen to the protein structure and calculate the charge. The three‐dimensional chemical structure was downloaded from PubChem. After setting the receptors' sizes, ligand molecules and Grid Box through the AutoGrid section of the software and the Nice Level to 20, the Number of GA Runs for virtual screening of molecular docking was selected as 50. Then, the optimal conformational affinity score of the docking was calculated. Each molecular docking result was repeated at least three times until the output was stable.

### 2.7 Experimental Verification

#### 2.7.1 H9C2 Cell Grouping and Modelling

After 72 h of culture, the supernatant was removed from the H9C2 cell culture dish, which was then washed twice with PBS and replenished with sugar‐free DMEM. Subsequently, the H9C2 cells were subjected to a 6‐h anoxic culture in an anaerobic incubator (95% N_2_ and 5% CO_2_) before replacing the medium with low‐sugar complete medium containing 10% FBS. Then, the cells were cultured under normoxic conditions (95% air and 5% CO_2_) at 37°C for 6 h to induce an oxygen–glucose deprivation/reoxygenation (OGD/R) model [16].

H9C2 cells were placed in 96‐well plates and randomly divided into five groups with three replicated wells in each group. The groups included a normal control group (NC), an OGD/R model group (OGD/R), an OGD/*R* + CHSSC‐treated serum group (OGD/*R* + CHSSC), an OGD/*R* + Z‐VAD‐FMK group (OGD/*R* + Z‐VAD) and an OGD/*R* + disulfiram (DSF) group (OGD/*R* + DSF). The OGD/*R* + CHSSC group was administered an optimal concentration‐treated serum of CHSSC determined by the CCK‐8 assay. The OGD/*R* + Z‐VAD and OGD/*R* + DSF group was treated with 20 μmol/L Z‐VAD‐FMK [17] and 10 μmol/L DSF [18], respectively (Z‐VAD‐FMK is an inhibitor that can irreversibly inhibiting the activation of caspase and thereby blocking cell apoptosis; DSF can inhibits GSDMD pore formation, IL‐1β secretion and inflammasome‐mediated pyroptosis in cells).

#### 2.7.2 Determination of Optimal Treated Serum Concentration Using CCK‐8 Assay

H9C2 cells were inoculated into 96‐well plates and each group had three replicate wells. Treated serum and non‐treated serum with concentrations of 0%, 5%, 10%, 15%, 20%, 25% and 30% were added, respectively. The blank group was only cell‐free medium. After a 24‐h culture, 10‐μL CCK‐8 solution was added to the medium. Following further culture at 37°C and 5% CO_2_ for 4 h, the optical density (OD) at 450 nm was measured by microplate reader. Cell survival rate was calculated based on the provided formula from the CCK‐8 kit, and the optimal intervention concentration of CHSSC‐treated serum was determined based on the survival rate of treated cells.

#### 2.7.3 Animal Grouping and Modelling

After adaptive feeding for 1 week, 50 rats were randomly divided into sham operation group (Sham), myocardial ischaemia–reperfusion model group (Model), CHSSC low‐dose‐treated group (CHSSC‐L, 0.22 g/kg/day), CHSSC medium‐dose treated group (CHSSC‐M, 0.43 g/kg/day) and CHSSC high‐dose‐treated group (CHSSC‐H, 0.86 g/kg/day). The Sham and Model groups were given the same amount of distilled water by gavage, and each group was administered the indicated treatment for seven consecutive days.

The rats were anaesthetised after 1 h of final administration, and the ECG electrodes were placed to monitor the changes of ST segment of lead II during ischaemia–reperfusion. The trachea was opened and connected to a ventilator, then the skin was cut lengthwise at the left edge of the sternum, and exposing the heart and separating the pericardium. The left anterior descending coronary artery was ligated using a needle inserted from the lower edge of the left atrial appendage. A significant elevation of the ST segment in lead II, a high T wave and darkened colour of the heart surface below the ligation line indicated a successful ligation. Reperfusion was performed for 120 min after 30 min of myocardial ischaemia. The anaesthesia and thoracotomy for the Sham group were the same as the Model group but without ligation. Rats were excluded from the study if they exhibited abnormal ECG before ligation, failed coronary artery ligation or no observable reperfusion, excessive bleeding or stopped heart beat and breath for more than 30 s.

#### 2.7.4 ELISA

After 120 min of reperfusion, 3–4 mL rat blood sample was collected through the abdominal aorta, and serum was obtained by centrifugation. cTnI, CK‐MB and LDH levels were measured by commercial ELISA kits. All the tests were performed strictly according to the manufacturer's instructions.

#### 2.7.5 Haematoxylin and Eosin (H&E) Staining

The left ventricle tissue was cut and fixed with 4% paraformaldehyde for 24 h. After the fixation, the tissue was washed with running water for 12 h. The tissue was dehydrated with gradient alcohol. Paraffin sections with a thickness of 4 μm were obtained using a microtome. Haematoxylin dye for 1–10 min and returned to blue with PBS; eosin staining was carried out for 1–5 min. Then, the section was dehydrated and sealed with neutral gum and observed under a microscope.

#### 2.7.6 Terminal Deoxynucleotidyl Transferase‐Mediated Nick End Labelling (TUNEL) Assay

##### 2.7.6.1 H9C2 Cells

The cell climbing slice was fixed with 4% paraformaldehyde for 30 min, followed by the addition of proteinase K working solution. Subsequently, 100 μL of 1× equilibration buffer was added. The required TdT enzyme incubation buffer was prepared in the proportions of 34 μL dd H_2_O, 10 μL 5× equilibration buffer, 5 μL FITC‐12‐dUTP Labelling Mix and 1 μL Recombinant TdT Enzyme. Next, 50 μL TdT incubation buffer was added and incubated at 37°C for 60 min. DAPI working solution was used for nuclear staining followed by sealing before observation under a fluorescence microscope.

##### 2.7.6.2 Cardiac Muscle Tissue in Rats

The heart tissue of rats was sectioned after dehydration, embedding and dewaxing. One hundred microliter proteinase K working solution was added to each sample and incubated at 37°C for 10 min. Following the instructions in the endogenous avidin‐biotin blocking kit and TUNEL assay kit, the sections were air dried and cover slipped with neutral resin. Observations and photography were conducted using a light microscope.

#### 2.7.7 Flow Cytometry Assay

After digestion with pancreatic enzymes without EDTA, the cells were washed twice with PBS. FAM‐YVAD‐FMK was added to suspend the cells, followed by the addition of propidium iodide and mixing well. The mixture was incubated at room temperature in the dark for 1 h. Finally, flow cytometry was used for the detection of cell pyroptosis rate.

#### 2.7.8 Western Blot Assay

RIPA lysis buffer was added to heart tissue and thoroughly homogenised until no visible tissue chunks remained. Briefly, to 200 μL of the protein supernatant, 50 μL of 5× loading buffer was added and boiled in water for 5 min, and then quickly cooled in an ice box for later use. The gel was electrophoresed at a constant voltage and the proteins were transferred onto a PVDF membrane at a constant current of 300 mA for different durations: PI3K, p‐PI3K: 100 min; caspase 8: 90 min; p53, p‐p53, AKT and p‐AKT: 80 min; caspase 1: 70 min; caspase3/7, β‐actin and GAPDH: 60 min; IL‐1β: 50 min; IL‐18: 45 min; and Bax: 40 min. The primary antibody was diluted in 1× PBST at the following ratios: caspase 7/8 at 1:500; caspase3, IL‐1β, p‐AKT and p‐PI3K at 1:1000; caspase 1, IL‐18, Bax, p53, p‐p53 and PI3K at 1:2000; AKT, β‐actin and GAPDH at 1:5000. HRP‐labelled secondary antibodies, goat anti‐Mouse IgG (H + L) and goat anti‐Rabbit IgG (H + L) were diluted to 1:5000 in 1 × PBST and incubated with the membrane at room temperature for 90 min. The membrane was incubated with ECL chemiluminescent solution and imaged on a gel imaging system.

### 2.8 Statistical Analysis

All statistical analyses were performed using SPSS29.0.2.0 software (IBM Corporation, Armonk, NY, USA). All data were presented as mean ± standard deviation (SD). One‐way ANOVA was used to compare differences among three or more groups, and post hoc Fisher's least significant difference (LSD) test or Dunnett's test was used for individual group comparisons. *p* < 0.05 were considered to indicate statistically significant differences.

## 3 Results

### 3.1 Serum Pharmaco‐Chemistry Analysis of CHSSC

**FIGURE 1:**
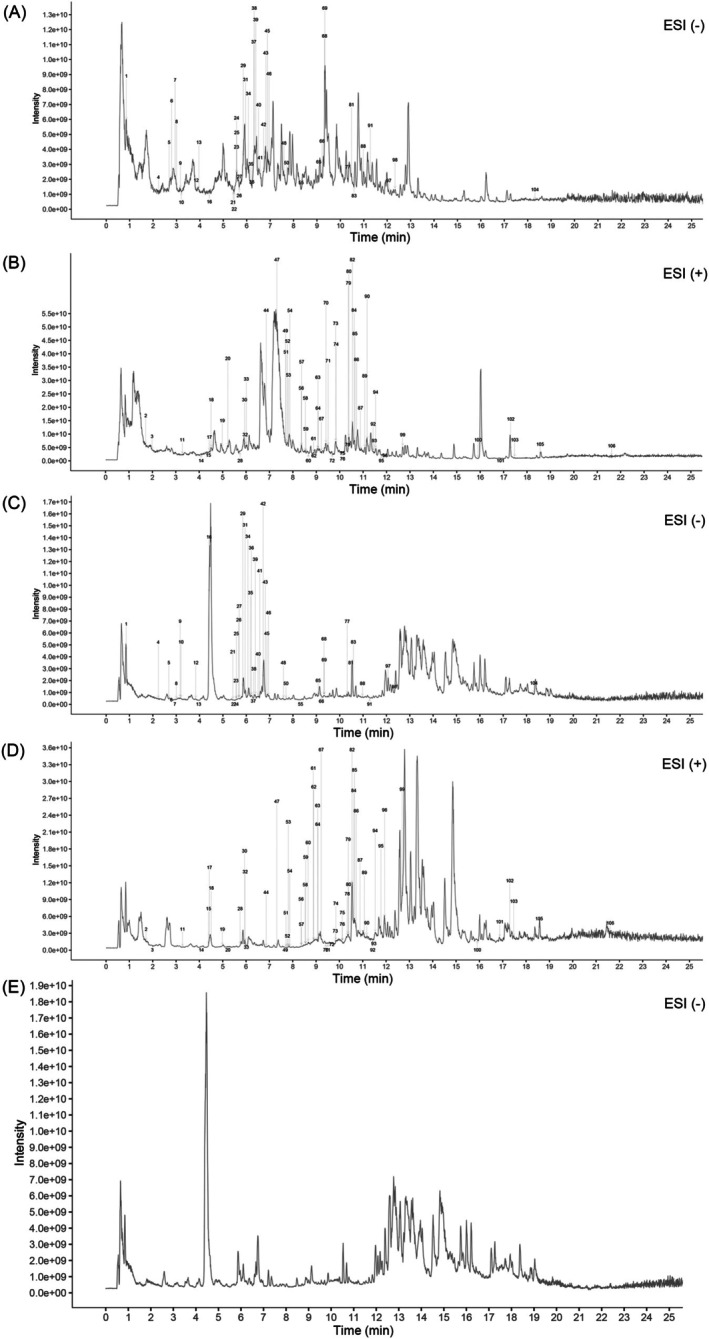
Total ion chromatograms (TIC) from CHSSC, treated serum and non‐treated serum. (A) TIC of CHSSC in negative ion mode. (B) TIC of CHSSC in positive ion mode. (C) TIC of treated serum in negative ion mode. (D) TIC of treated serum in positive ion mode. (E) TIC of non‐treated serum in negative ion mode.

**FIGURE 2:**
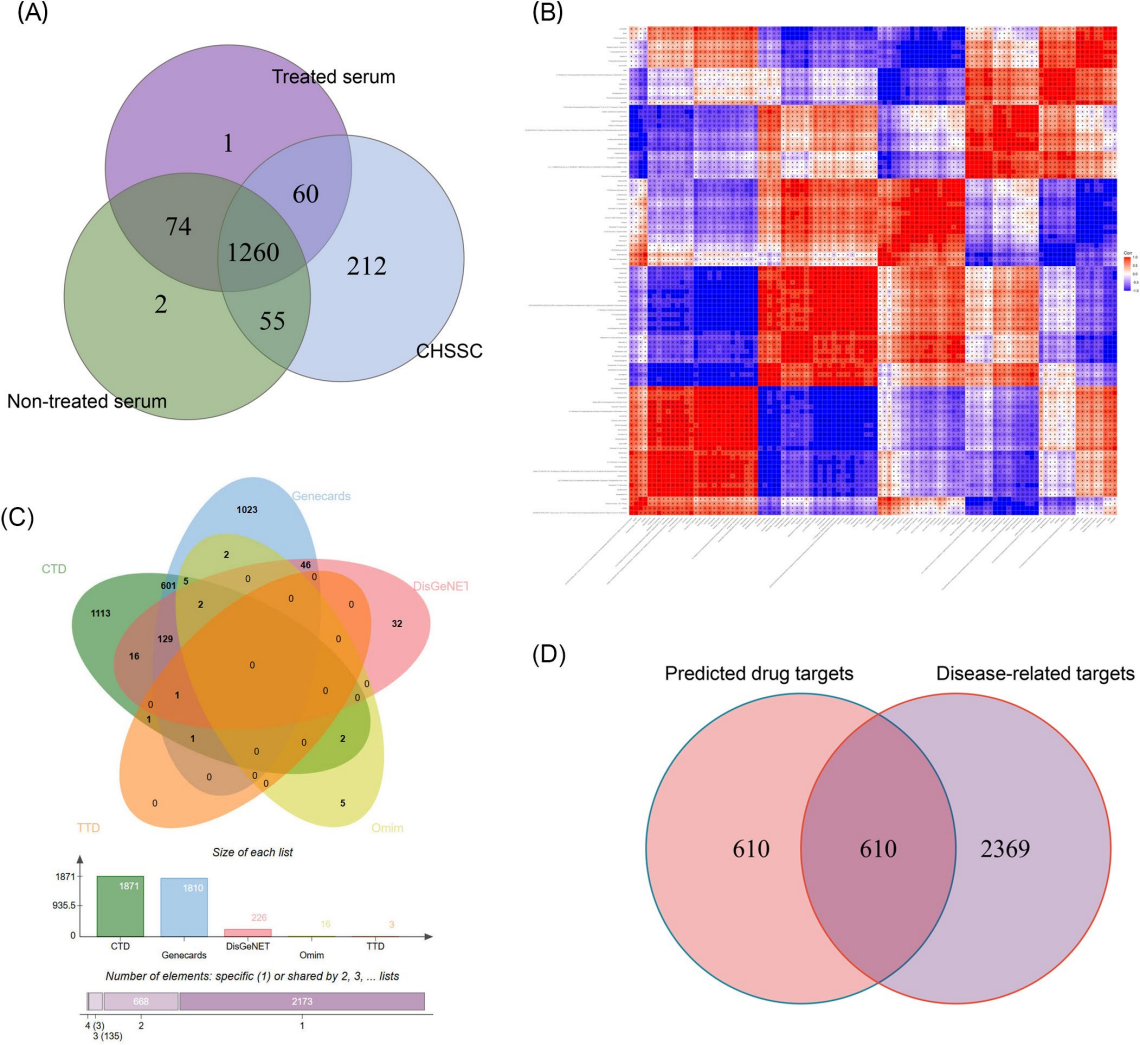
Relationship of active compounds of CHSSC and targets prediction. (A) Venn diagram of the relationship of CHSSC, treated serum and non‐treated serum. (B) The correlation analysis of the compounds of CHSSC absorbed into the bloodstream. (C) Venn diagram of the intersection of disease‐related targets in different database. (D) Venn diagram of the intersection of predicted targets and disease‐related targets.

The CHSSC, non‐treated serum and treated serum groups were detected by UHPLC‐QE‐MS assay. The total ion chromatograms (TIC) are presented in Figure 1. A total of 1587 compounds were detected in CHSSC, 1395 compounds in treated serum of CHSSC and 1391 compounds in non‐treated serum. The relationship between them is shown in a Venn diagram (Figure 2A). A total of 106 compounds were identified in the treated samples. These compounds were classified into three grades—I, II and III—based on blood absorption characteristics. Grade I (*n* = 60) comprised compounds present in both CHSSC and treated serum but not in the non‐treated serum, representing prototype components of CHSSC absorbed into the bloodstream. Grade II (*n* = 45) included compounds found in all three samples, with significantly higher content in treated serum than non‐treated serum, indicating potential absorption into the bloodstream. Grade III (*n* = 1) comprised compounds exclusively present in treated serum, possibly representing the metabolites of CHSSC. These 106 compounds can be primarily categorised as flavonoids, terpenoids, alkaloids, lipids, organic acids, coumarins and phenols. The detailed mass spectrometry data, including retention time, molecular mass, chemical formula, fragments and blood level are presented in Table S1.

As shown in Figure 2B, correlation analysis was conducted on these 106 compounds to quantify their close interrelationships. The horizontal and vertical axes in the figure represent the compounds and colour‐coded blocks at various positions indicate correlation coefficients between corresponding compounds. Red signifies a positive correlation, whereas blue indicates a negative correlation, with darker shades representing stronger correlations. Additionally, non‐significant correlations are denoted by crosses.

### 3.2 Network Pharmacology Analysis

#### 3.2.1 Target Prediction of CHSSC

After screening, a total of 50 effective and active compounds from CHSSC (including 26 Grade I components and 24 Grade II components) were identified and renumbered as CHSSC1‐50 (refer to Table S2). Through these compounds, we predicted 412 targets in the TCMSP database, 1825 targets in the Swiss database and 1601 targets in the SEA database. A total of 1220 predicted drug targets were obtained through database comparison and removal of duplicate targets. Myocardial ischaemia–reperfusion‐related targets were systematically searched across various databases, and 2979 targets were obtained after integration, including CTD (1871), Genecards (1810), DisGeNET (226), OMIM (16) and TTD (3). A total of 610 targets were identified through the intersection of the predicted drug targets and disease‐related targets. The Venn diagram of the intersection of disease‐related targets in different databases is shown in Figure 2C, and the intersection of predicted targets and disease‐related targets is shown in Figure 2D.

#### 3.2.2 PPI Network

**FIGURE 3:**
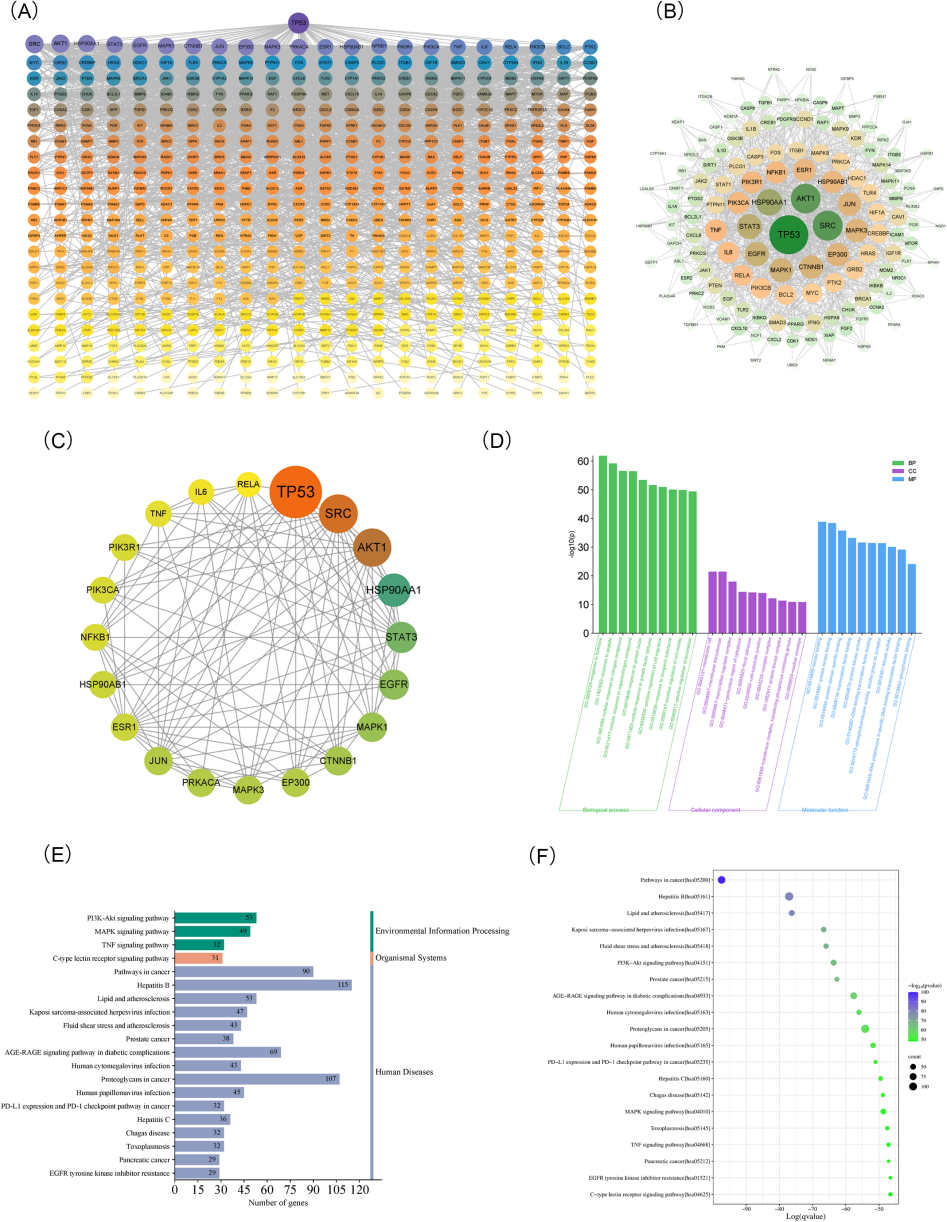
The core targets of CHSSC and its GO and KEGG analysis information in the treatment of MIRI. (A) The PPI network diagram depicts the interaction of target proteins. (B) Core targets and their correlations network diagram. (C) Top 20 targets and their relationship network diagram. (D) Histogram of the top 10 GO‐enriched analysis items for BP, CC and MF. (E) The secondary classification of action pathways. (F) Bubble diagram of the top 20 core action pathways.

The PPI network diagram depicts the interaction of target proteins of CHSSC in the treatment of MIRI (Figure 3A). Subsequently, a total of 138 core targets were further identified and their correlations are presented in Figure 3B. Network topology analysis of the PPI network revealed that the top 20 hub targets of CHSSC for treating MIRI were TP53, SRC, AKT1, HSP90AA1, STAT3, EGFR, MAPK1, CTNNB1, JUN, EP300, MAPK3, PRKACA, ESR1, HSP90AB1, NF‐κB1, PIK3R1, PIK3CA, TNF, IL6 and RELA. Detailed information of these targets is shown in Table 2, and their relationship is illustrated in Figure 3C.

**TABLE 2:** Information of the top 20 core targets of CHSSC for the treatment of MIRI.

No.	Name	Degree	Betweenness	Closeness	Network	Indicator significance
1	TP53	86	35,232.473	0.039377697	53.67005	p53 signalling pathway
2	SRC	61	17,388.256	0.039185185	32.49787	Na/K‐ATPase/Src signalling pathway
3	AKT1	60	12,063.967	0.039298713	27.892927	PI3K/Akt signalling pathway
4	HSP90AA1	51	10,040.748	0.03916488	21.963495	Common regulatory target of multiple programmed cell death (PCD)
5	STAT3	47	9263.19	0.039252058	20.954166	JAK2/STAT3 signalling pathway
6	EGFR	45	7348.1865	0.03914459	20.427343	EGFR signalling pathway
7	MAPK1	44	6659.1904	0.03917938	19.870604	MAPK signalling pathway
8	CTNNB1	43	5864.6226	0.039115645	19.16996	Wnt/β‐catenin signalling pathway
9	JUN	42	9794.825	0.039272457	21.642551	MAPK signalling pathway
10	EP300	42	12,689.55	0.03917068	20.681978	Activity regulation of TP53
11	MAPK3	42	6328.3125	0.03915618	18.598948	MAPK signalling pathway
12	PRKACA	42	20,781.79	0.03901468	7.990641	PKC signalling pathway
13	ESR1	40	6569.6123	0.03920842	18.094074	Oestrogen receptor
14	HSP90AB1	40	4515.136	0.039008923	18.028164	Common regulatory target of multiple programmed cell death (PCD)
15	NFKB1	39	6517.069	0.039124325	15.07537	NF‐κB signalling pathway
16	PIK3R1	39	1808.749	0.038805753	22.825024	PI3K/Akt signalling pathway
17	PIK3CA	39	2528.1152	0.038954344	21.843246	PI3K/Akt signalling pathway
18	TNF	38	8386.895	0.0389888	17.508863	Inflammatory factor
19	IL6	37	9274.895	0.038951475	18.36502	Inflammatory factor
20	RELA	36	3882.8877	0.03904347	14.489948	NF‐κB signalling pathway

#### 3.2.3 GO and KEGG Analysis

Four hundred and fifty‐eight BP items, 112 CC items and 123 MF items were identified through GO functional enrichment analysis. The top 10 enriched items for BP, CC and MF were selected to generate a histogram and bubble diagram illustrating the analysis results. As shown in Figure 3D, the functions involved encompass responses to hormones and peptides, as well as cellular responses to nitrogen and organonitrogen compounds. Additionally, they include responses to growth factors and cellular reactions to stimuli from growth factors, among others. The action pathways of CHSSC mainly involve Human Diseases (16 pathways), Environmental Information Processing (three pathways) and Organismal Systems (one pathway). The secondary classification is illustrated in Figure 3E. Enrichment analysis identified that pathways related to cancer, Hepatitis B and lipid metabolism and atherosclerosis were the most significantly enriched. These pathways were prominently clustered in the upper‐left quadrant of the bubble plot (Figure 3F), which serves as a visualisation highlighting the top 20 core pathways associated with MIRI.

#### 3.2.4 Docking Analysis

**FIGURE 4:**
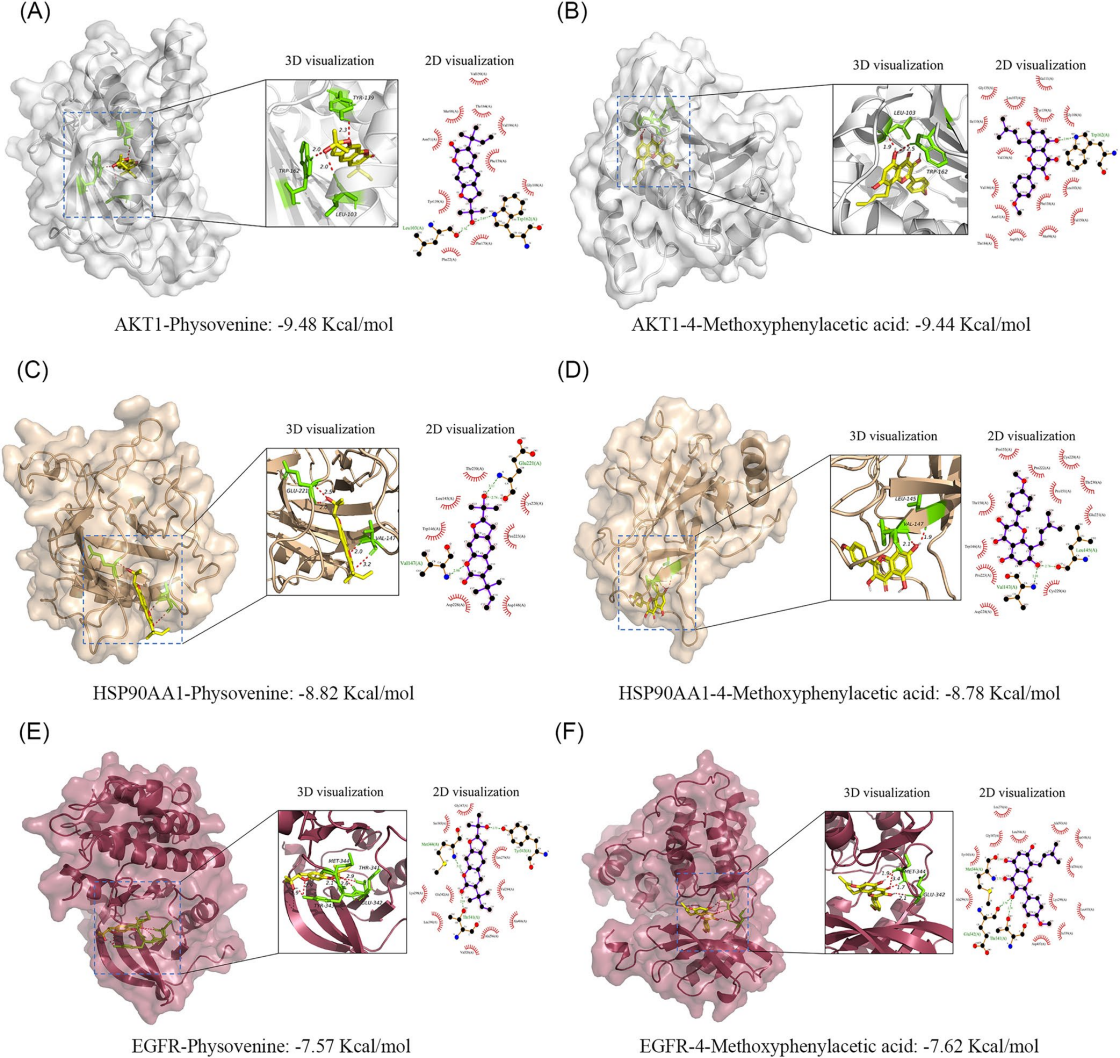
Molecular docking of CHSSC's active compounds with target proteins. (A) Physovenine and AKT1. (B) 4‐Methoxyphenylacetic acid and AKT1. (C) Physovenine and HSP90AA1. (D) 4‐Methoxyphenylacetic acid and HSP90AA1. (E) Physovenine and EGFR. (F) 4‐methoxyphenylacetic acid and EGFR.

Semi‐flexible molecular docking was performed on the top six core targets of the CHSSC degree value, namely TP53, SRC, AKT1, HSP90AA1, STAT3 and EGFR protein receptors. A lower binding energy corresponds to a higher affinity between the receptor and ligand, resulting in a more stable conformation. Our results shown that there were 35 receptor‐ligand combinations exhibiting a binding energy of ≤ −4.25 kcal/mol, 31 with a binding energy of ≤ −5.0 kcal/mol and 10 with a binding energy of ≤ −7.0 kcal/mol, suggesting that the majority of receptor‐ligand combinations displayed favourable binding capabilities. Physovenine exhibits strong binding affinity with three target proteins, among which AKT1 showed the strongest binding affinity with two hydrogen bonding sites. 4‐Methoxyphenylacetic acid showed strong binding affinity with four target proteins, among which AKT1 displayed the strongest binding activity with two hydrogen binding sites. The docking binding energies for the top 10 receptor‐ligand combinations are presented in Table 3. Among these 10 receptor‐ligand combinations, the receptors AKT1, HSP90AA1, EGFR and TP53 indicated that these targets played an important role in the treatment of MIRI. The results of the molecular docking simulations for the representative active compounds Physovenine and 4‐methoxyphenylacetic acid with key targets AKT1, HSP90AA1 and EGFR are illustrated in Figure 4.

**TABLE 3:** Binding energy information of active compounds of CHSSC and target proteins.

Transferrin receptor	PDB ID	Molecule ligand	Binding energy (kcal/mol)
AKT1	2UZR	Physovenine	−9.48
AKT1	2UZR	4‐Methoxyphenylacetic acid	−9.44
HSP90AA1	3R91	Physovenine	−8.82
HSP90AA1	3R91	4‐Methoxyphenylacetic acid	−8.78
EGFR	2RGP	4‐Methoxyphenylacetic acid	−7.62
EGFR	2RGP	Physovenine	−7.57
AKT1	2UZR	6‐(1,1‐Dimethylallyl)‐2‐(1‐hydroxy‐1‐methylethyl)‐2,3‐dihydro‐7H‐furo[3,2‐G]chromen‐7‐one	−7.48
HSP90AA1	3R91	6‐(1,1‐Dimethylallyl)‐2‐(1‐hydroxy‐1‐methylethyl)‐2,3‐dihydro‐7H‐furo[3,2‐G]chromen‐7‐one	−7.46
AKT1	2UZR	Icaritin	−7.30
TP53	3ZME	4‐Methoxyphenylacetic acid	−7.30

### 3.3 Effect of CHSSC on Markers of Myocardial Injury in MIRI Rat

**FIGURE 5:**
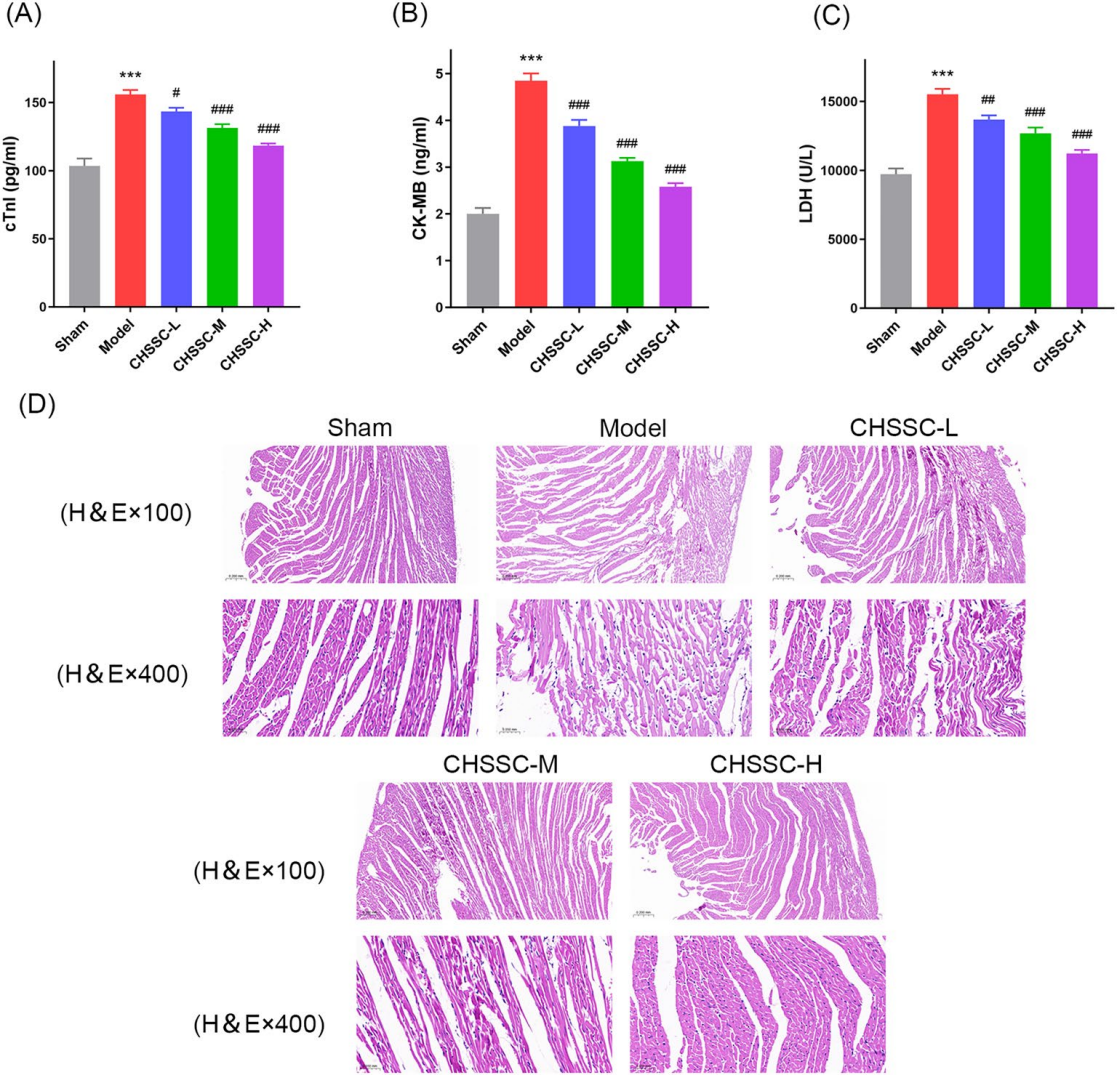
Effect of CHSSC on markers of myocardial injury and histopathological examination in MIRI rats. (A) Effect of CHSSC on cTnI in MIRI rats. (B) Effect of CHSSC on CK‐MB in MIRI rats. (C) Effect of CHSSC on LDH in MIRI rats. (D) Effect of CHSSC on histopathological examination (H&E staining ×100 and ×400) in MIRI rats. *n* = 3, *p < 0.05, **p < 0.01, ***p < 0.001 versus the Sham operation group; ^#^ *p* < 0.05, ^##^ *p* < 0.01, ^###^ *p* < 0.001 versus model group.

As shown in Figure 5A–C, serum levels of cTnI, CK‐MB and LDH were significantly increased in the Model group compared to the Sham group; however, following the intervention of CHSSC, these levels showed a significant decrease compared to the Model group. Among the CHSSC low‐, medium‐ and high‐dose treated groups, the reduction was most pronounced in the high‐dose‐treated group.

### 3.4 Effect of CHSSC on Histopathological Examination in MIRI Rats

As illustrated in Figure 5D, HE staining showed that the myocardium of the Sham group exhibited a normal tissue structure without oedema, lesions or neutrophil infiltration. In contrast to the Sham group, the Model group displayed significant myocardial injury characterised by myocardial structural disarray, interstitial oedema and haemorrhage, as well as numerous vacuoles. Compared with the Model group, the CHSSC medium‐ and high‐dose‐treated group showed morphological improvement, whereas the high‐dose‐treated group demonstrated a more significant alleviating effect.

### 3.5 Effect of CHSSC on Myocardial Cell Apoptosis

**FIGURE 6:**
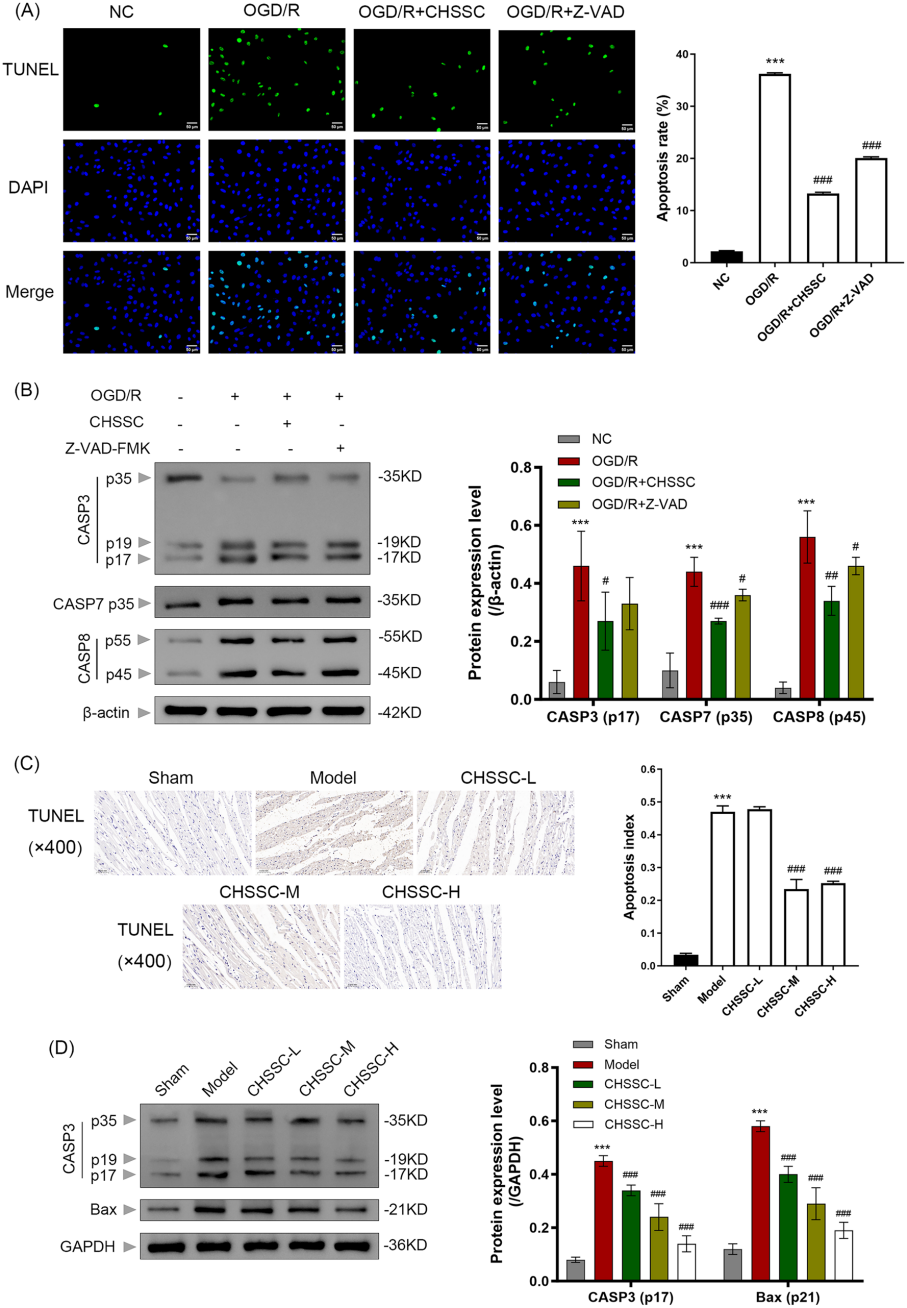
Effect of CHSSC on apoptosis in OGD/R H9C2 cells and MIRI rats. (A) Apoptosis rate of H9C2 cells determined by TUNEL. (B) Western blotting of apoptosis‐related proteins CASP3, CASP7 and CASP8. (C) Apoptosis rate of rat cardiomyocytes determined by TUNEL. (D) Western blotting of apoptosis‐related proteins CASP3 and Bax. *n* = 3, *p < 0.05, **p < 0.01, ***p < 0.001 versus NC group or Sham operation group; ^#^ *p* < 0.05, ^##^ *p* < 0.01, ^###^ *p* < 0.001 versus OGD/R group or model group.

As shown in Table 4, the survival rate of H9C2 cells remained stable when the concentration of CHSSC‐treated serum was no more than 15%. However, cell viability declined when the concentration exceeded 20%. Therefore, 15% CHSSC‐treated serum was selected for subsequent experiments. As shown in Figure 6A, green fluorescence was considered a positive signal. The apoptosis rate of the OGD/R model group was significantly higher than that of the control group; however, following treatment with CHSSC and Z‐VAD‐FMK, the apoptosis rate decreased. Western blot analysis (Figure 6B) showed that the expression levels of caspase‐3 (p17) and caspase‐7/8 significantly increased in the OGD/R model group. The protein expression level decreased after the intervention of CHSSC and Z‐VAD‐FMK.

**TABLE 4:** The mean OD value and survival rate of H9C2 cells after treatment with different concentrations of CHSSC‐treated serum.

Groups	Blank	0% treated serum group	5% treated serum group	10% treated serum group	15% treated serum group	20% treated serum group	25% treated serum group	30% treated serum group
Mean OD value	0.2214	1.1423	1.3103	1.2678	1.2185	1.1337	1.0512	0.9459
Survive rate	—	—	118.24%	113.64%	108.28%	99.07%	90.11%	78.68%

As shown in Figure 6C, the TUNEL staining results of rat myocardia showed that compared with the Sham group, the proportion of TUNEL‐positive cells (nuclear staining to brown) in the MIRI Model group was significantly increased. Following intervention, the CHSSC group exhibited a reduction in the proportion of TUNEL‐positive cells compared to the model group. As shown in Figure 6D, the expressions of Bax and caspase‐3 (p17) in the myocardial tissue of rats in the MIRI model group significantly increased compared with the Sham group, while they decreased after CHSSC treatment. These results indicated that CHSSC could inhibit myocardial apoptosis induced by ischaemia–reperfusion.

### 3.6 Effect of CHSSC on Myocardial Cell Pyroptosis

**FIGURE 7:**
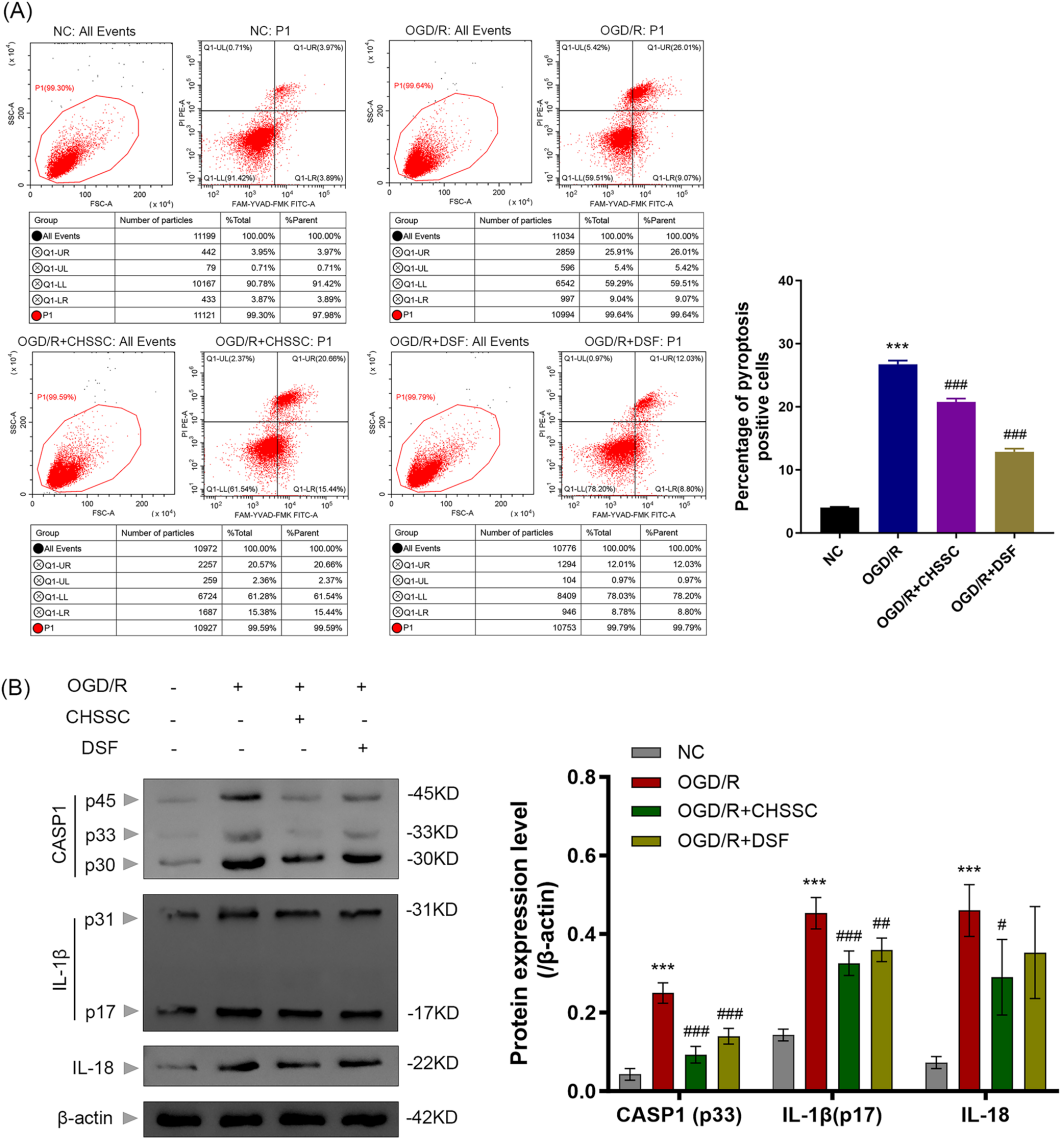
Effect of CHSSC on pyroptosis in OGD/R H9C2 cells. (A) Pyroptosis rate of H9C2 cells determined by flow cytometry assay. (B) Western blotting of pyroptosis‐related proteins CASP1, IL‐1β and IL‐18. *n* = 3, *p < 0.05, **p < 0.01, ***p < 0.001 versus NC group; ^#^ *p* < 0.05, ^##^ *p* < 0.01, ^###^ *p* < 0.001 versus OGD/R group.

As shown in Figure 7A, the pyroptosis rate of H9C2 cells subjected to OGD/R significantly increased compared to the normal control group. In contrast, treatment with CHSSC and DSF resulted in a decrease in the pyroptosis rate of H9C2 cells when compared to the OGD/R model group. Western blot results (Figure 7B) showed that OGD/R increased the activation of caspase‐1, a pivotal protein within the pyroptosis pathway and the expression of IL‐1β and IL‐18. These alterations were ameliorated following treatment with CHSSC and DSF. Collectively, these results suggest that CHSSC has potential therapeutic effects on reducing pyroptosis in H9C2 cells post‐OGD/R.

### 3.7 Effect of CHSSC on the PI3K/AKT/p53 Signalling Pathway in MIRI Rats

**FIGURE 8:**
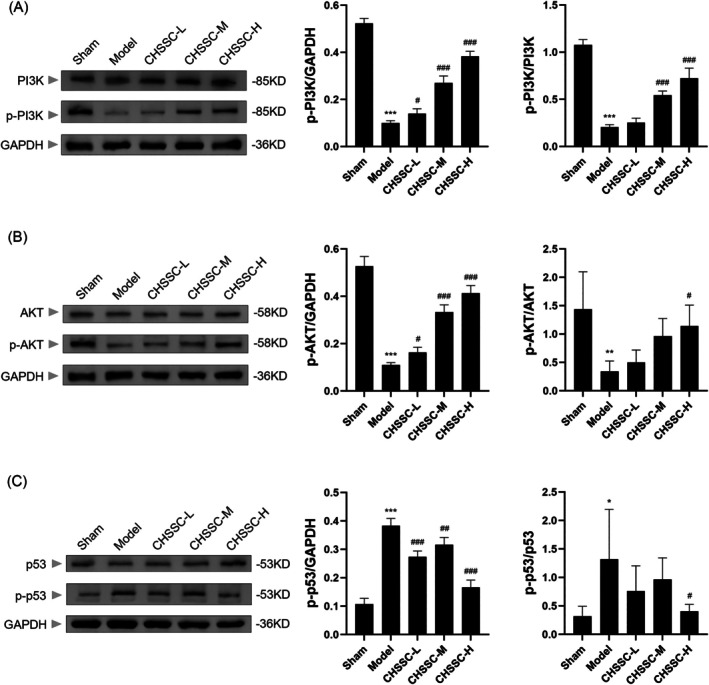
Effect of CHSSC on PI3K/AKT/p53 signalling pathway in myocardial tissue of MIRI rats. (A) Western blotting of PI3K, p‐PI3K. (B) Western blotting of AKT, p‐AKT. (C) Western blotting of p53, p‐p53. *n* = 3, *p < 0.05, **p < 0.01, ***p < 0.001 versus the Sham operation group; ^#^ *p* < 0.05, ^##^ *p* < 0.01, ^###^ *p* < 0.001 versus model group.

As illustrated in Figure 8, the expression levels of p‐PI3K and p‐AKT in the myocardium of rats from the MIRI model group were significantly decreased compared to those in the Sham group, whereas the expression level of p‐p53 was increased. Following CHSSC treatment, a marked increase in p‐PI3K and p‐AKT expression was observed, alongside a decrease in the expression level of p‐p53. The protein expression changes were most pronounced in the high‐dose CHSSC group. These results indicate that CHSSC may exert a protective effect following myocardial ischaemia–reperfusion by regulating the PI3K/AKT/p53 signalling pathway.

## 4 Discussion

Current strategies for addressing MIRI include ischaemic preconditioning [19], mitochondrial modulation [20] and nanomedicine applications [21], among others. Although these interventions offer promising therapeutic avenues, the development of safe and effective pharmacological agents continues to face substantial challenges. Research on Chinese herbal compounds presents novel opportunities and insights for treating MIRI. Chinese medicine boasts a complex composition of multiple components targeting various pathways, but the unclear mechanisms of action of these complex formulations are also a limitation of this treatment system. Nevertheless, serum pharmaco‐chemistry analysis can help to elucidate the fundamental chemical constituents and bioavailability aspects of Chinese herbal compounds to better understand their mechanisms. Furthermore, network pharmacology enriches our comprehension of its multi‐targeted and multi‐pathway action networks.

According to TCM theory, MIRI is classified as ‘chest impediment’ related to ‘qi stagnation’ and ‘phlegm and blood stasis’. The recommended treatment for this condition involves ‘moving qi’, ‘resolving stasis’ and ‘activating blood’ [22]. CHSSC is a traditional Chinese herbal compound formulation consisting of eight herbs based on the understanding of chest impediment in TCM. Our preliminary research indicates that it can effectively improve MIRI [15]. In this study, UHPLC‐QE‐MS was employed to analyse a total of 1587 compounds present in CHSSC, revealing its complex composition. However, 106 compounds—including flavonoids, terpenes, alkaloids, lipids and organic acids—were absorbed into the bloodstream of rats via ingestion and gastrointestinal absorption, suggesting their potential role as active compounds of CHSSC. Many compounds present in CHSSC that are absorbed into the bloodstream have demonstrated protective effects against MIRI. For instance, quercetin mitigates MIRI by inhibiting oxidative stress, preventing apoptosis and reducing calcium overload [23]; nobiletin could alleviate MIRI in type 2 diabetic rats through the inhibition of ferroptosis [24]; and baicalin prevents against MIRI by suppressing ACSL4‐controlled ferroptosis [25]. Even so, many compounds of CHSSC have yet to be studied for their activity against MIRI, such as tetrahydropersin, dihydrokaempferol and oxyberberine. Furthermore, various compounds may interact synergistically to form a network that collectively targets relevant pathways. Through network pharmacology analysis, we can further analyse the core compounds and key targets of the ‘active compounds‐targets‐pathways‐diseases’ network. This approach provides a huge research space for discovering effective compounds and targets for MIRI treatment, as well as exploring synergistic therapies involving multiple compounds and targets.

The network pharmacology analysis of CHSSC shows that its core targets are TP53, SRC, AKT1, HSP90AA1, STAT3 and so on. These targets are implicated in the regulation of oxidative stress responses, inflammatory processes, apoptosis mechanisms and various facets of programmed cell death. Through various pathways, p53 mediates the regulation of apoptosis, programmed necrosis, autophagy, ferroptosis and oxidative stress in MIRI. Consequently, pharmacological agents targeting p53‐related therapeutic pathways are anticipated to serve as promising options for MIRI treatment [26]. The activation of STAT3 signalling in MIRI promotes cellular recovery by attenuating the generation of ROS, alleviating calcium overload and reducing apoptosis [27]. Through target analysis, it is indicated that the mechanism of CHSSC intervention in MIRI may involve regulating multiple signalling pathways, such as p53, PI3K/AKT, JAK2/STAT3, EGFR and MAPK, to achieve multi‐target and multi‐pathway regulation of MIRI and exert myocardial protective effects.

In the KEGG database, pathways are classified into seven main categories: Metabolism, Genetic Information Processing, Environmental Information Processing, Cellular Processes, Organismal Systems, Human Diseases and Drug Development. The pathways associated with CHSSC primarily involve the Human Diseases category, the Environmental Information Processing category and the Organismal Systems category. Pathway enrichment analyses indicated that human disease signalling pathways such as lipid and atherosclerosis (ranked third) and fluid shear stress and atherosclerosis (ranked fifth) are closely related to MIRI. Interestingly, the role of AGE‐RAGE signalling pathway (ranked eight) has been substantiated by many studies on diabetic complications such as neuropathy, renal injury and cardiovascular disease [28, 29], particularly in diabetic MIRI [30]. MAPK signalling and TNF signalling, which fall under the Environmental Information Processing category, are mechanistically implicated in MIRI through their regulation of inflammatory responses, oxidative stress and apoptosis [31, 32], however, their relatively low enrichment significance in our bioinformatics analysis suggests these pathways may represent critical yet underappreciated therapeutic targets of CHSSC in MIRI management.

Through the establishment of the ‘CHSSC‐active compounds‐core target‐pathway‐MIRI’ network, we found that the majority of top 10 active components were flavonoids such as *quercetin*, *icaritin*, *flavokavain A* and *nobiletin*, indicating that flavonoids may play a key role in myocardial protective. Nevertheless, research on compounds from Chinese herbal medicine shows that flavonoids, alkaloids and coumarins can all alleviate MIRI [23, 33, 34]. The findings from these studies corroborated the network pharmacology analysis presented in this paper, suggesting that CHSSC is well‐positioned to emerge as a prominent Chinese herbal compound for the treatment of MIRI in the foreseeable future. It is crucial to emphasise that, while network pharmacology and molecular docking predictions supply theoretical support for the multi‐target effects of the herbal formulation, and functional experiments offer indirect evidence for target modulation, direct validation of interaction kinetics between bioactive compounds and core targets through in vitro binding assays (e.g., surface plasmon resonance [SPR], isothermal titration calorimetry [ITC]) remains necessary for mechanistic confirmation. Such experimental strategies have been effectively employed in the determination and validation of binding affinities between monomers of traditional Chinese herbs and target proteins [35, 36, 37]. Building upon these studies, we will integrate quantitative interaction analysis to confirm and quantify the strength of interactions between the bioactive compounds and their predicted targets.

By integrating serum pharmaco‐chemistry with network pharmacology analysis, we can achieve a more comprehensive understanding of the active constituents and interaction networks of traditional Chinese herbal compounds. This approach enables us to systematically study the mechanisms underlying their holistic actions from a network perspective, thereby facilitating the identification of potentially promising compounds and therapeutic targets. Further in‐depth exploration and additional experimental validation are essential for studies on CHSSC, which will also outline directions for future research endeavours.

The Bcl‐2 protein family serves as a crucial regulator of apoptosis, significantly contributing to the process of cell death [38]. Upon binding of the BH3 protein to its domain at the N‐terminal Bax activation site, cytosolic Bax is activated and undergoes a conformational change. When Bax is conformationally active, it translocates to the outer mitochondrial membrane, where it assembles into oligomeric structures that form Bax pores. These oligomeric complexes, in conjunction with other regions of Bax, create higher order pores that permeabilise the mitochondrial outer membrane, facilitating the release of cytochrome C and other apoptotic factors, ultimately triggering cellular apoptosis [39]. During MIRI, the expression of Bax and caspase‐3 is upregulated, whereas Bcl‐2 is inhibited. Pharmacological inhibition of Bax has been shown to prevent cardiomyocyte death and alleviate MIR and is regarded as a promising therapeutic target [40, 41]. Our results showed that Bax and caspase‐3 were overexpressed in MIRI model rats but decreased after CHSSC intervention, suggesting that Bax and caspase‐3 may direct or downstream targets of CHSSC. In vitro experiments showed that intervention of CHSSC‐treated serum decreased the apoptosis rate of H9C2 cells and reduced the expression of apoptosis‐related proteins in the OGD/R model group. We also observed pyroptosis in H9C2 cells. CHSSC could likely reduce the rate of pyroptosis in the OGD/R model group of H9C2 cells. There exists a crosstalk among cell apoptosis, pyroptosis, and necroptosis, collectively referred to as PANoptosis [42]. A recent study has demonstrated the presence of PANoptosis‐like cell death in ischaemia–reperfusion injury of retinal neurons [43]. PANoptosis is considered associated with multiple cardiovascular diseases including atherosclerosis, myocardial infarction, aortic aneurysm and dissection, and it may represent a significant therapeutic target [44]. However, research on PANoptosis in MIRI currently lacks corresponding experimental evidence. This study indirectly suggests that PANoptosis‐like cell death may exist in MIRI, and CHSSC may target PANoptosis to exert its effect, but this requires further experimental verification. Additionally, while H9C2 cells and rats provided a practical model for this study, their translational potential is inherently limited by species‐specific differences. To address this limitation, we plan to construct MIRI models utilising iPSC‐derived cardiomyocytes or AC16 to validate the expression and functional consistency of key molecular targets and further assess the correlation between the findings of this study and human pathology.

Multiple signalling pathways are involved in the regulation of Bax, including PI3K/AKT, MEK1‐ERK1/2 and JAK2/STAT3 [41]. Network pharmacology analysis of CHSSC indicates that signalling pathways such as PI3K/AKT, JAK2/STAT3 and NF‐κB are all its core pathways of action. The downstream targets of the PI3K/AKT signalling pathway include GSK3, eNOS, BAD, IKK, MDM2 and p53, which are involved in regulating the cell cycle, inflammatory response and apoptosis. During MIRI, the activation of PI3K/AKT signalling cascade can further modulate the downstream p53 pathway and exert myocardial protection by regulating processes such as cell apoptosis, oxidative stress, autophagy activities and inhibiting mitochondrial dysfunction [26, 45]. We explored the mechanism of CHSSC on MIRI through in vivo experiments conducted on rats. Our results showed that CHSSC can increase the phosphorylation expression levels of PI3K and AKT, and reduce that of p53. Subsequently, p53 regulates downstream targets of cell apoptosis like Bax and caspase‐3, thereby inhibiting apoptotic processes and mitigating cardiac injury induced by ischaemia and reperfusion.

Nevertheless, a comprehensive understanding of the overall mechanism by which CHSSC ameliorates MIRI necessitates more experimental works, given the complexity components and the diversity of its targets. This includes elucidating the specific role of individual compounds, verifying the effects on autophagy, ferroptosis, PANoptosis or achieving a more thorough comprehension of the involved targets or pathways. Appling a systematic or holistic approach rather than a fragmented one may prove more beneficial in deciphering or researching the mechanisms underlying Chinese herbal compounds. Alternatively, we can obtain a clearer and more integrated understanding of the action network associated with these Chinese herbal compounds by employing AI [46, 47].

## 5 Conclusions

This study expands upon our prior research on CHSSC and provides a comprehensive analysis of the constituents in treated serum to clarify its substance basis and potential active compounds. Additionally, we employed network pharmacology to analyse the core compounds, targets and pathways involved in CHSSC's intervention for MIRI and conducted molecular docking analyses between the core compounds and their targets; however, it should be noted that these computationally predicted targets still require further validation through confirmatory binding assays. Furthermore, in vivo rat experiments and in vitro analysis on H9C2 cells validated the pharmacological effects of CHSSC and its core targets and pathways obtained from previous network pharmacology analysis, showing that CHSSC inhibited apoptosis and alleviated MIRI mainly through the PI3K/AKT/p53 signalling pathway.

## Author Contributions

**Weisong Wang:** conceptualization (lead), formal analysis (lead), investigation (lead), writing – original draft (lead). **Zengyu Zhang:** data curation (equal), formal analysis (equal), investigation (equal), software (equal). **Rongzhen Liu:** writing – original draft (equal). **Yu Zheng:** writing – original draft (equal). **Yaqi Hu:** writing – review and editing (equal). **Xia Li:** formal analysis (equal), visualization (equal). **Zihao Shen:** methodology (equal). **Hengyou Yuan:** methodology (equal), software (equal). **Jianhe Liu:** conceptualization (equal), funding acquisition (equal), project administration (equal), supervision (equal), writing – review and editing (equal).

## Conflicts of Interest

The authors declare no conflicts of interest.

## Supporting information


**Table S1:** jcmm70666‐sup‐0001‐TableS1.docx.

**Table S2:** jcmm70666‐sup‐0002‐TableS2.docx.

## Data Availability

The data that support the findings of this study are available from the corresponding author upon reasonable request.
