# Supplementary material for: Exploration of the Potential Bioactive Compounds and Functional Mechanism of Chaihu Sanshen Capsule in Ameliorating Myocardial Ischaemia–Reperfusion Injury: A Serum Pharmaco‐Chemistry With Network Pharmacology Analysis
Source: J Cell Mol Med. 2025 Jul 28;29(14):e70666. doi: 10.1111/jcmm.70666 (PMC12303846; doi:10.1111/jcmm.70666)
Supplement: Supplementary file 1 — Table S1: jcmm70666‐sup‐0001‐TableS1.docx. [file JCMM-29-e70666-s002.docx]

Supplementary table 1 Analysis of components of CHSSC in serum

| NO. | t_R_ (s) | Type | Extraction mass (Da) | Error (ppm) | Formula | Identification | ms2Adduct | Fragments | Blood level | Class |
| --- | --- | --- | --- | --- | --- | --- | --- | --- | --- | --- |
| 1 | 51.52 | - | 344.0396 | 1.2252 | C_10_H_12_N_5_O_7_P | Cyclic GMP | [M-H]- | 150.0416;344.0399;133.0161;92.6864;89.0245 | I | Purine nucleotide |
| 2 | 102.93 | + | 179.0337 | 1.4918 | C_9_H_6_O_4_ | 7,8-Dihydroxycoumarin | [M+H]+ | 133.0281;179.0328;95.0489;107.0487;123.0438 | I | Coumarins |
| 3 | 119.14 | + | 139.0867 | 1.8564 | C_7_H_10_N_2_O | 3-Ethyl-2-methoxypyrazine | [M+H]+ | 121.0754;139.0862;109.0761;111.0441;95.0487 | II | Pyrazine |
| 4 | 133.92 | - | 415.1718 | 2.8248 | C_23_H_28_O_7_ | Methyl (1R,4aS,9S,10S)-10-acetyloxy-5,9-dihydroxy-1,4a-dimethyl-2-oxo-7-propan-2-yl-10,10a-dihydro-9H-phenanthrene-1-carboxylate | [M-H]- | 253.1192;124.0409;325.1397;94.0301;101.0247 | I | Terpenoids |
| 5 | 161.06 | - | 180.0665 | 2.7414 | C_9_H_11_NO_3_ | L-Tyrosine | [M-H]- | 163.0405;181.0503;135.0453;119.0506;72.9933 | II | Organic acid |
| 6 | 167.62 | - | 461.1297 | 0.6559 | C_19_H_26_O_13_ | [(2R,3S,4S,5R,6R)-6-[(2S,3S,4S,5R)-3,4-dihydroxy-2,5-bis(hydroxymethyl)oxolan-2-yl]oxy-3,4,5-trihydroxyoxan-2-yl]methyl 4-hydroxybenzoate | [M-H]- | 137.0238;461.1211;92.6847;121.0287;89.0244 | I | Polyketides |
| 7 | 176.50 | - | 165.0556 | 2.1875 | C_9_H_10_O_3_ | 4-Methoxyphenylacetic acid | [M-H]- | 165.0549;59.0139;93.0342;121.0652;119.0506 | II | Phenolic acids |
| 8 | 180.28 | - | 153.0192 | 1.0885 | C_7_H_6_O_4_ | Gentisic acid | [M-H]- | 109.0290;153.0186;108.0212;151.0030;95.0140 | II | Xanthones |
| 9 | 189.99 | - | 459.1146 | 0.8925 | C_19_H_24_O_13_ | Parishin E | [M-H]- | 111.0086;459.1105;87.0089;173.0091;92.6839 | I | Phenols |
| 10 | 192.32 | - | 255.0876 | 2.4253 | C_12_H_16_O_6_ | Phenyl-beta-glucopyranoside | [M-H]- | 59.0138;255.0860;165.0548;72.9932;179.0343 | I | Organic oxygen compounds |
| 11 | 196.45 | + | 263.1389 | 0.1815 | C_14_H_18_N_2_O_3_ | Physovenine | [M+H]+ | 70.0650;263.1403;177.1004;87.0437;245.1295 | II | Alkaloids |
| 12 | 230.37 | - | 461.1669 | 0.1878 | C_19_H_28_O_10_ | Phenylethyl primeveroside | [M+FA-H]- | 179.0697;461.1678;89.0243;167.0353;59.0139 | I | Alkaloids |
| 13 | 237.45 | - | 293.1239 | 0.2818 | C_14_H_18_N_2_O_5_ | Glutamylphenylalanine | [M-H]- | 293.1232;131.0709;59.0139;89.0244;71.0138 | II | Miscellaneous |
| 14 | 244.88 | + | 296.1660 | 16.9105 | C_19_H_21_NO_2_ | Nuciferine | [M+H]+ | 296.1609;162.1017;278.1454;69.0335;70.0649 | II | Alkaloids |
| 15 | 263.46 | + | 312.1588 | 0.5757 | C_14_H_21_N_3_O_5_ | Leonurine | [M+H]+ | 312.1580;107.0486;58.0651;267.0994;297.1348 | II | Alkaloid |
| 16 | 264.26 | - | 593.1500 | 1.5924 | C_27_H_30_O_15_ | 5,7-dihydroxy-2-(4-hydroxyphenyl)-6,8-bis[3,4,5-trihydroxy-6-(hydroxymethyl)oxan-2-yl]chromen-4-one | [M-H]- | 593.1505;353.0679;383.0780;473.1104;297.0758 | I | Flavonoids |
| 17 | 265.78 | + | 305.1128 | 32.1190 | C_16_H_16_O_6_ | Isosaxalin; Heraclenol | [M+H]+ | 305.1029;287.0929;92.6643;288.03207;243.0353 | I | Coumarins |
| 18 | 270.48 | + | 245.0955 | 1.9861 | C_10_H_16_N_2_O_3_S | Biotin | [M+H]+ | 227.0843;245.0965;97.0385;167.0519;166.0685 | II | Alkaloid |
| 19 | 298.93 | + | 281.1387 | 2.5567 | C_15_H_20_O_5_ | Crispolide | [M+H]+ | 217.1232;189.0914;245.1176;263.1278;221.1160 | II | Lipid |
| 20 | 313.33 | + | 237.1850 | 4.3169 | C_15_H_24_O_2_ | Curcumol | [M+H]+ | 237.1575;151.1116;133.1008;219.14600;59.0492 | II | Terpenoids |
| 21 | 325.45 | - | 195.0662 | 1.24136 | C_10_H_12_O_4_ | Dihydroferulic acid | [M-H]- | 160.8419;195.8101;136.0527;195.0667;92.6871 | II | Organic acid |
| 22 | 327.87 | - | 579.2073 | 1.15194 | C_28_H_36_O_13_ | S(8-8)S hexoside | [M-H]- | 417.1583;181.0503;255.0658;166.0265;579.1732 | I | Lignans |
| 23 | 333.67 | - | 435.1298 | 0.3971 | C_21_H_24_O_10_ | Phloretin-2'-O-glucoside | [M-H]- | 273.0779;435.1312;167.0354;92.6855;255.0668 | I | Flavonoids |
| 24 | 333.97 | - | 401.1090 | 0.11698 | C_16_H_20_O_9_ | Gentiopicroside | [M+FA]- | 401.1105;151.0398;249.0630;201.0204;113.0242 | I | Terpenoids |
| 25 | 334.52 | - | 469.13418 | 1.75782 | C_21_H_26_O_12_ | Plumieride | [M-H]- | 99.0454;143.0343;145.0292;325.0899;187.0399 | I | Terpenoids |
| 26 | 340.72 | - | 533.13092 | 0.1427 | C_25_H_26_O_13_ | 5,7-dihydroxy-2-(4-hydroxyphenyl)-6,8-bis(3,4,5-trihydroxyoxan-2-yl)chromen-4-one | [M-H]- | 533.1369;443.0975;353.0673;383.0784;191.0553 | I | Flavonoids |
| 27 | 341.81 | - | 463.0887 | 0.5818 | C_21_H_20_O_12_ | Quercetin-3-O-galactoside | [M-H]- | 300.0298;463.0849;301.0333;271.0231;255.0288 | I | Flavonoids |
| 28 | 344.73 | + | 465.1023 | 0.5918 | C_21_H_20_O_12_ | Isoquercitrin | [M+H]+ | 303.0512;85.0282;61.0284;91.03897;97.0278 | I | Flavonoids |
| 29 | 351.05 | - | 563.14075 | 0.4373 | C_26_H_28_O_14_ | Apiin | [M-H]- | 269.0447;563.1415;270.0475;92.6839;353.0679 | I | Flavonoids |
| 30 | 356.16 | + | 257.0807 | 1.1067 | C_15_H_12_O_4_ | Liquiritigenin | [M+H]+ | 257.0811;137.0231;147.0446;119.0492;239.0707 | II | Flavonoids |
| 31 | 357.12 | - | 431.0986 | 0.9217 | C_21_H_21_O_10_ | Pelargonidin-3-O-glucoside | [M-2H]- | 268.0384;269.0446;431.0951;92.6830;432.0989 | II | Miscellaneous |
| 32 | 357.77 | + | 433.1133 | 0.6213 | C_19_H_22_O_10_ | aloenin | [M+Na]+ | 271.0594;433.1086;313.0708;321.0956;320.0882 | II | Phenols |
| 33 | 360.70 | + | 299.1756 | 2.0129 | C_18_H_22_N_2_O_2_ | 2,9-Dimethyl-2,9-diazatricyclo[10.2.2.25,8]octadeca-5,7,12,14,15,17-hexaene-3,10-diol, 9CI | [M+H]+ | 299.1756;200.1057;162.0918;202.1236;271.1780 | I | Organic nitrogen compounds |
| 34 | 364.18 | - | 449.1087 | 0.6089 | C_21_H_22_O_11_ | Eriodictyol-7-O-glucoside | [M-H]- | 287.0554;244.0372;259.0612;59.0137;449.2006 | I | Flavonoids |
| 35 | 370.73 | - | 144.0453 | 1.9797 | C_9_H_7_NO | 1H-Indole-3-carboxaldehyde | [M-H]- | 144.0454;145.0493;92.6863;116.0499;151.0946 | II | Indoles |
| 36 | 372.91 | - | 463.0885 | 1.0196 | C_21_H_21_O_12_ | Delphinidin-3-O-beta-glucopyranoside | [M-2H]- | 463.0760;300.0296;161.0243;301.0325;113.0245 | I | Flavonoids |
| 37 | 378.18 | - | 433.1133 | 1.5948 | C_21_H_22_O_10_ | Naringenin-7-O-glucoside | [M-H]- | 113.0243;433.1122;85.0297;152.9969;257.0834 | II | Flavonoids |
| 38 | 379.19 | - | 623.1624 | 0.9502 | C_28_H_32_O_16_ | Narcissoside | [M-H]- | 315.0504;623.1608;314.0451;299.0221;300.02813 | I | Flavonoids |
| 39 | 383.10 | - | 447.0928 | 0.4173 | C_21_H_20_O_11_ | Kaempferol-3-O-glucoside | [M-H]- | 447.0951;284.0318;255.0288;227.03474;285.0370 | I | Flavonoids |
| 40 | 390.40 | - | 143.1076 | 2.9208 | C_16_H_31_NaO_4_ | Valproic acid | [M-H]- | 143.1072;92.6846;71.0137;144.0453;122.0682 | II | Organic acid |
| 41 | 394.60 | - | 301.0716 | 1.2586 | C_16_H1_4_O_6_ | Hesperetin | [M-H]- | 301.1662;73.0294;283.1567;209.1171;150.0331 | II | Flavonoids |
| 42 | 403.36 | - | 353.0877 | 0.7388 | C_16_H_18_O_9_ | Cryptochlorogenic acid | [M-H]- | 173.0458;179.0342;191.0552;135.0453;353.0871 | I | Phenols |
| 43 | 408.77 | - | 415.1977 | 1.6305 | C_19_H_30_O_7_ | 3,5,5-Trimethyl-4-[3-[3,4,5-trihydroxy-6-(hydroxymethyl)oxan-2-yl]oxybut-1-enyl]cyclohex-2-en-1-one | [M+HCOO]- | 415.1925;92.6881;179.0571;89.0245;71.0137 | I | Miscellaneous |
| 44 | 411.74 | + | 397.1131 | 0.3506 | C_16_H_22_O_10_ | Swertiamarin | [M+Na]+ | 193.0491;397.1088;92.6627;132.5665;70.0650 | II | Terpenoids |
| 45 | 412.90 | - | 477.1042 | 0.4400 | C_22_H_23_O_12_ | Petunidin-3-O-beta-glucopyranoside | [M-2H]- | 77.1074;315.0485;314.0457;116.9288;92.6847 | II | Flavonoids |
| 46 | 417.09 | - | 561.1618 | 0.4028 | C_27_H_30_O_13_ | 7-[3-[(2R,3R,4R)-3,4-dihydroxy-4-(hydroxymethyl)oxolan-2-yl]oxy-4,5-dihydroxy-6-(hydroxymethyl)oxan-2-yl]oxy-3-(4-methoxyphenyl)chromen-4-one | [M-H]- | 267.0676;252.0421;268.0722;85.0292;168.0059 | I | Flavonoids |
| 47 | 439.24 | + | 291.0857 | 1.0874 | C_15_H_14_O_6_ | Isoplumericin | [M+H]+ | 291.0878;245.0817;189.0909;217.0866;199.0759 | I | Terpenoids |
| 48 | 455.37 | - | 407.1347 | 0.7994 | C_20_H_24_O_9_ | Nodakenin | [M-H]- | 119.0506;407.1317;163.0404;99.0453;80.9650 | I | Coumarins |
| 49 | 461.04 | + | 303.0496 | 1.2474 | C_15_H_10_O_7_ | Quercetin | [M+H]+ | 303.0512;267.1572;153.0184;229.0479;137.0597 | I | Flavonoids |
| 50 | 461.99 | - | 445.0777 | 0.6032 | C_21_H_18_O_11_ | Baicalin | [M-H]- | 269.0446;445.0673;113.0246;268.0382;267.0312 | II | Flavonoids |
| 51 | 462.64 | + | 195.1745 | 2.5557 | C_13_H_22_O | Oxidized Latia luciferin | [M+H]+ | 177.1637;121.1007;195.1218;107.0852;95.0848 | III | Miscellaneous |
| 52 | 466.47 | + | 151.0391 | 0.9599 | C_8_H_6_O_3_ | 4-Hydroxyphthalide | [M+H]+ | 151.0394;121.0287;123.0438;93.0337;67.0541 | I | Miscellaneous |
| 53 | 468.50 | + | 265.1432 | 1.0431 | C_15_H_20_O_4_ | Eudesmane naphthofuran | [M+H]+ | 219.1365;247.1323;265.1922;205.1218;201.1271 | II | Terpenoids |
| 54 | 472.40 | + | 461.1084 | 0.9872 | C_22_H_20_O_11_ | Wogonoside | [M+H]+ | 285.0742;270.0507;461.1071;85.0282;151.0394 | II | Flavonoids |
| 55 | 499.37 | - | 163.0763 | 1.5833 | C_10_H_12_O_2_ | 4-Isopropylbenzoic acid | [M-H]- | 162.8392;127.8708;159.8599;125.8728;135.0810 | II | Lipid |
| 56 | 501.58 | + | 241.0494 | 1.4711 | C_14_H_8_O_4_ | 1,2-Dihydroxy anthraquinone | [M+H]+ | 241.0496;125.0591;220.5806;97.9847;194.4354 | I | Quinones |
| 57 | 502.47 | + | 447.1284 | 0.8408 | C_22_H_22_O_10_ | Biochanin-7-O-glucoside | [M+H]+ | 285.0742;270.0509;447.1241;123.0437;229.0872 | II | Flavonoids |
| 58 | 512.15 | + | 313.1550 | 0.0112 | C_17_H_25_ClO_3_ | Chloropanaxydiol | [M+H]+ | 313.1540;285.1617;239.1186;271.1439;211.1210 | II | Miscellaneous |
| 59 | 513.26 | + | 385.12834 | 0.8960 | C_21_H_20_O_7_ | Uralene | [M+H]+ | 385.1277;313.0698;285.0752;367.1186;92.6644 | I | Flavonoids |
| 60 | 519.68 | + | 359.1493 | 0.7998 | C_20_H_22_O_6_ | Matairesinol | [M+H]+ | 137.0596;341.1372;131.0493;291.1029;163.0748 | I | Lignans |
| 61 | 533.18 | + | 223.1695 | 2.1476 | C_14_H_22_O_2_ | 2,6-Di-tert-butyl-1,4-benzenediol | [M+H]+ | 165.1264;195.1365;223.1318;149.1315;109.1010 | II | Aromatic compounds |
| 62 | 534.07 | + | 325.1069 | 0.3935 | C_19_H_16_O_5_ | R-10-Hydroxywarfarin | [M+H]+ | 325.1068;281.1167;211.0394;251.1083;263.1074 | I | Flavonoids |
| 63 | 544.08 | + | 293.2110 | 0.1715 | C_18_H_28_O_3_ | (2'E,4'Z,7'Z,8E)-Colnelenic acid | [M+H]+ | 275.1991;293.2102;107.0853;81.0699;67.0541 | II | Lipid |
| 64 | 544.52 | + | 313.1434 | 1.0741 | C_19_H_20_O_4_ | Honyudisin | [M+H]+ | 269.1521;313.1437;171.0806;271.0974;199.0761 | II | Coumarins |
| 65 | 544.64 | - | 329.1390 | 0.0734 | C_19_H_22_O_5_ | Gibberellin | [M-H]- | 241.1598;329.1400;285.1506;92.6846;173.0976 | II | Terpenoids |
| 66 | 553.17 | - | 941.5129 | 0.0885 | C_48_H_78_O_18_ | Soybean saponin fraction B1 | [M-H]- | 941.4919;941.5193;92.6855;101.02466;71.0137 | I | Terpenoids |
| 67 | 553.24 | + | 943.5255 | 4.7540 | C_48_H_78_O_18_ | Buddlejasaponin IVb | [M+H]+ | 85.0283;92.6635;437.3432;419.3295;455.3482 | II | Terpenoids |
| 68 | 559.63 | - | 281.0454 | 1.3277 | C_16_H_10_O_5_ | Pseudobaptigenin | [M-H]- | 281.0448;253.0504;92.6854;151.6349;209.4558 | II | Flavonoids |
| 69 | 560.14 | - | 829.4947 | 1.5490 | C_42_H_72_O_13_ | Ginsenoside Rg2 | [M+HCOO]- | 783.4902;783.5110;59.0139;475.3836;101.0247 | I | Terpenoids |
| 70 | 564.67 | + | 370.1284 | 0.9726 | C_20_H_19_NO_6_ | Ochratoxin B | [M+H]+ | 163.0392;370.1258;92.6652;352.1543;220.0967 | I | Coumarins |
| 71 | 570.20 | + | 269.0806 | 1.3597 | C_16_H_12_O_4_ | Tectochrysin | [M+H]+ | 269.0808;254.0584;213.0899;237.0532;253.0508 | II | Flavonoids |
| 72 | 580.57 | + | 247.0965 | 1.9500 | C_14_H_14_O_4_ | Corticrocin | [M+H]+ | 229.0872;247.1807;187.0393;185.0964;143.0491 | I | Lipid |
| 73 | 589.46 | + | 352.1178 | 0.4286 | C_20_H_17_NO_5_ | Oxyberberine | [M+H]+ | 352.1166;308.0919;324.1246;280.0960;336.0851 | I | Alkaloid |
| 74 | 590.78 | + | 311.1271 | 0.4499 | C_19_H_18_O_4_ | Moracin C | [M+H]+ | 311.1249;267.1372;252.1148;265.1229;268.1423 | I | Phenols |
| 75 | 607.40 | + | 339.1222 | 6.5643 | C_20_H_18_O_5_ | Glyceollin III | [M+H]+ | 339.1208;92.6635;69.0694;95.08477;347.1133 | I | Flavonoids |
| 76 | 607.40 | + | 271.1331 | 0.4356 | C_17_H_18_O_3_ | 4-tert-Butylphenyl salicylate | [M+H]+ | 271.1312;229.0840;253.1204;187.0395;197.0588 | II | Phenols |
| 77 | 619.25 | - | 287.0562 | 0.9003 | C_15_H_12_O_6_ | Dihydrokaempferol | [M-H]- | 109.0299;92.6863;213.6517;178.9615;145.5822 | II | Flavonoids |
| 78 | 620.19 | + | 403.1381 | 2.1293 | C_21_H_22_O_8_ | Nobiletin | [M+H]+ | 403.1405;373.0878;388.1131;327.0849;355.0797 | I | Flavonoids |
| 79 | 622.39 | + | 797.4676 | 3.0313 | C_42_H_68_O_14_ | Soyasaponin III | [M+H]+ | 441.3744;141.0180;423.3634;92.6669;85.0282 | I | Lipid |
| 80 | 623.61 | + | 245.1174 | 1.5041 | C_15_H_16_O_3_ | Linderalactone | [M+H]+ | 227.1072;245.1186;212.0817;92.6635;185.0585 | I | Terpenoids |
| 81 | 628.78 | - | 313.1097 | 4.0474 | C_18_H_18_O_5_ | Flavokawain A | [M-H]- | 313.1083;225.1644;80.9649;269.1532;268.2009 | I | Flavonoids |
| 82 | 632.52 | + | 225.1849 | 0.2576 | C14H24O2 | 5Z,8Z-tetradecadienoic acid | [M+H]+ | 189.1632;207.1741;133.1009;165.1647;95.0848 | I | Organic acid |
| 83 | 635.23 | - | 357.1344 | 1.1505 | C20H22O6 | (+)-Pinoresinol | [M-H]- | 357.1367;329.2315;92.6864;299.1296;211.1333 | II | Lignans |
| 84 | 636.95 | + | 455.2067 | 0.6427 | C26H30O7 | Obacunone | [M+H]+ | 179.0341;455.3575;313.0682;437.1987;303.1596 | I | Terpenoids |
| 85 | 638.91 | + | 423.3254 | 1.4451 | C27H44O2 | Calcidiol | [M+H]+ | 423.2406;109.1010;95.0856;203.1442;92.6644 | I | Lipid |
| 86 | 643.07 | + | 450.2486 | 0.8357 | C24H32O7 | Schizandrin | [M+NH4]+ | 415.2124;384.1903;92.6627;352.1104;369.1685 | I | Lignans |
| 87 | 653.13 | + | 301.1069 | 0.3723 | C17H16O5 | Odoriflavene | [M+H]+ | 123.0437;301.1092;165.0539;179.0711;137.0594 | I | Phenols |
| 88 | 658.55 | - | 367.1290 | 27.3623 | C21H20O6 | Icaritin | [M-H]- | 367.1198;309.0395;297.0408;284.0310;203.0714 | II | Flavonoids |
| 89 | 664.25 | + | 327.1227 | 0.8208 | C19H18O5 | 1,7-Dihydroxy-3-methoxy-2-prenylxanthone | [M+H]+ | 309.1101;265.1229;223.0748;236.0838;247.1107 | II | Flavonoids |
| 90 | 670.47 | + | 787.4604 | 3.2361 | C42H68O12 | Saikosaponin E | [M+Na]+ | 787.4520;92.6635;127.0388;109.0286;85.0282 | I | Terpenoids |
| 91 | 676.90 | - | 339.1231 | 0.2698 | C20H20O5 | Flavanone base + 3O, 1Prenyl | [M-H]- | 339.1250;151.0033;187.1128;92.6872;107.0138 | I | Flavonoids |
| 92 | 685.80 | + | 358.2007 | 0.7925 | C21H27NO4 | Laudanoside | [M+H]+ | 358.1989;92.6661;114.8679;310.0761;243.1001 | I | Alkaloid |
| 93 | 688.63 | + | 281.0807 | 1.1669 | C15H14O4 | Luvangetin | [M+Na]+ | 281.0810;239.0323;253.0862;81.0693;92.6652 | I | Coumarins |
| 94 | 692.16 | + | 425.1964 | 0.9358 | C25H28O6 | Kushenol F | [M+H]+ | 165.0177;289.1433;183.0289;283.0581;425.1952 | I | Flavonoids |
| 95 | 706.84 | + | 329.2683 | 1.0604 | C19H36O4 | Avocadene 4-acetate | [M+H]+ | 329.2660;69.0696;83.0855;57.0701;55.0544 | I | Lipid |
| 96 | 716.42 | + | 455.2073 | 0.5515 | C26H30O7 | (1R,2R,4S,7R,8S,12R)-7-(furan-3-yl)-1,8,12,17,17-pentamethyl-3,6,16-trioxapentacyclo[9.9.02,4.02,8.012,18]icos-13-ene-5,15,20-trione | [M+H]+ | 179.0348;319.1519;455.3505;197.0455;121.1003 | I | Terpenoids |
| 97 | 724.10 | - | 313.1446 | 1.3933 | C19H22O4 | 6-(1,1- 6-(1, 1-Dimethylallyl)-2-(1-hydroxy-1-methylethyl)-2,3-dihydro-7H-furo[3,2]chromen-7-one | [M-H]- | 313.1451;283.1343;255.1399;295.1323;285.1512 | II | Coumarins |
| 98 | 740.10 | - | 501.3221 | 0.1636 | C30H46O6 | Medicagenic acid | [M-H]- | 501.3262;439.3249;92.6847;55.7050;502.3285 | II | Terpenoids |
| 99 | 761.38 | + | 297.1481 | 0.3776 | C19H20O3 | Cryptotanshinone | [M+H]+ | 297.1495;251.1421;279.1379;254.0930;282.1251 | I | Terpenoids |
| 100 | 954.52 | + | 285.2214 | 1.4153 | C20H28O | Retinal | [M+H]+ | 285.2200;267.2112;129.0698;157.1014;171.1168 | II | Lipid |
| 101 | 1012.11 | + | 331.2633 | 1.0296 | C22H34O2 | ent-16-Kauren-19-ol acetate | [M+H]+ | 331.2639;91.0538;105.0701;119.0848;81.0694 | II | Lipid |
| 102 | 1038.12 | + | 385.3312 | 0.6486 | C23H44O4 | Tetrahydropersin | [M+H]+ | 385.3283;57.0700;83.0488;71.0856;95.0855 | II | Lipid |
| 103 | 1048.31 | + | 427.3564 | 0.8529 | C29H46O2 | (6beta,22E)-6-Hydroxystigmasta-4,22-dien-3-one | [M+H]+ | 409.3454;427.3571;95.0848;92.6635;81.0693 | I | Terpenoids |
| 104 | 1098.66 | - | 279.2328 | 0.8357 | C18H32O2 | Linoleic acid | [M-H]- | 279.2306;280.2382;216.1946;189.8786;111.2987 | II | Lipid |
| 105 | 1113.60 | + | 130.0863 | 2.0971 | C6H11NO2 | Pipecolic acid | [M+H]+ | 84.0806;130.0858;70.0650;88.1117;74.0963 | I | Alkaloid |
| 106 | 1296.57 | + | 556.5301 | 2.0508 | C34H69NO4 | Armillaramide | [M+H]+ | 60.0442;556.5261;538.5201;282.2782;264.2686 | I | Lipid |

Note: Blood level I: ingredients that contained in CHSSC and medicated serum but not contained in non-medicated serum; II: ingredients that contained in CHSSC, medicated serum and non-medicated serum, but the content in medicated serum was significantly higher than non-medicated serum; III: ingredients that contained in medicated serum but not contained in CHSSC and non-medicated, it may be the metabolites of CHSSC.
