# Supplementary material for: Exploration of the Potential Bioactive Compounds and Functional Mechanism of Chaihu Sanshen Capsule in Ameliorating Myocardial Ischaemia–Reperfusion Injury: A Serum Pharmaco‐Chemistry With Network Pharmacology Analysis
Source: J Cell Mol Med. 2025 Jul 28;29(14):e70666. doi: 10.1111/jcmm.70666 (PMC12303846; doi:10.1111/jcmm.70666)
Supplement: Supplementary file 2 — Table S2: jcmm70666‐sup‐0002‐TableS2.docx. [file JCMM-29-e70666-s001.docx]

Supplementary table 2 Renumber information of active compounds of CHSSC after screening

| **NO.** | **Molecule Name** | **Canonical SMILES** | **Formula** | **Extraction mass (Da)** | **t_R_ (s)** | **ms2Adduct** |
| --- | --- | --- | --- | --- | --- | --- |
| CHSSC1 | (-)-Heraclenol | CC(C)(C(COC1=C2C(=CC3=C1OC=C3)C=CC(=O)O2)O)O | C_16_H_16_O_6_ | 305.1128 | 265.78 | [M+H]+ |
| CHSSC2 | (1R,2R,4S,7R,8S,12R)-7-(furan-3-yl)-1,8,12,17,17-pentamethyl-3,6,16-trioxapentacyclo[9.9.02,4.02,8.012,18]icos-13-ene-5,15,20-trione | CC1(C2CC(=O)C3(C(C2(C=CC(=O)O1)C)CCC4(C35C(O5)C(=O)OC4C6=COC=C6)C)C)C | C_26_H_30_O_7_ | 455.2072 | 716.42 | [M+H]+ |
| CHSSC3 | (5Z,8Z)-tetradeca-5,8-dienoic acid | CCCCCC=CCC=CCCCC(=O)O | C_14_H_24_O_2_ | 225.1849 | 632.52 | [M+H]+ |
| CHSSC4 | (6beta,22E)-6-Hydroxystigmasta-4,22-dien-3-one | CCC(C=CC(C)C1CCC2C1(CCC3C2CC(C4=CC(=O)CCC34C)O)C)C(C)C | C_29_H_46_O_2_ | 427.3564 | 1048.31 | [M+H]+ |
| CHSSC5 | (R)-10-Hydroxywarfarin | CC(=O)C(C(C1=CC=CC=C1)C2=C(C3=CC=CC=C3OC2=O)O)O | C_19_H_16_O_5_ | 325.1069 | 534.07 | [M+H]+ |
| CHSSC6 | 1,2-Dihydroxyheptadec-16-en-4-yl acetate | CC(=O)OC(CCCCCCCCCCCC=C)CC(CO)O | C_19_H_36_O_4_ | 329.2683 | 706.84 | [M+H]+ |
| CHSSC7 | 2,9-Dimethyl-2,9-diazatricyclo[10.2.2.25,8]octadeca-5,7,12,14,15,17-hexaene-3,10-diol, 9CI | CN1C(CC2=CC=C(C=C2)N(C(CC3=CC=C1C=C3)O)C)O | C_18_H_22_N_2_O_2_ | 299.1756 | 360.70 | [M+H]+ |
| CHSSC8 | 3,5,5-Trimethyl-4-[3-[3,4,5-trihydroxy-6-(hydroxymethyl)oxan-2-yl]oxybut-1-enyl]cyclohex-2-en-1-one | CC1=CC(=O)CC(C1C=CC(C)OC2C(C(C(C(O2)CO)O)O)O)(C)C | C_19_H_30_O_7_ | 415.1977 | 408.77 | [M+HCOO]- |
| CHSSC9 | Alizarin | C1=CC=C2C(=C1)C(=O)C3=C(C2=O)C(=C(C=C3)O)O | C_14_H_8_O_4_ | 241.0494 | 501.58 | [M+H]+ |
| CHSSC10 | Calcifediol | CC(CCCC(C)(C)O)C1CCC2C1(CCCC2=CC=C3CC(CCC3=C)O)C | C_27_H_44_O_2_ | 423.3254 | 638.91 | [M+H]+ |
| CHSSC11 | Corticrocin | C(=CC=CC=CC(=O)O)C=CC=CC=CC(=O)O | C_14_H_14_O_4_ | 247.0965 | 580.57 | [M+H]+ |
| CHSSC12 | Cryptotanshinone | CC1COC2=C1C(=O)C(=O)C3=C2C=CC4=C3CCCC4(C)C | C_19_H_20_O_3_ | 297.1481 | 761.38 | [M+H]+ |
| CHSSC13 | Flavokawain A | COC1=CC=C(C=C1)C=CC(=O)C2=C(C=C(C=C2OC)OC)O | C_18_H_18_O_5_ | 313.1097 | 628.78 | [M-H]- |
| CHSSC14 | Glyceollin III | CC(=C)C1CC2=CC3=C(C=C2O1)OCC4(C3OC5=C4C=CC(=C5)O)O | C_20_H_18_O_5_ | 339.1222 | 607.40 | [M+H]+ |
| CHSSC15 | Isoplumericin | CC=C1C2C3(C=CC4C3C(O2)OC=C4C(=O)OC)OC1=O | C_15_H_14_O_6_ | 291.0857 | 439.24 | [M+H]+ |
| CHSSC16 | Laudanosine | CN1CCC2=CC(=C(C=C2C1CC3=CC(=C(C=C3)OC)OC)OC)OC | C_21_H_27_NO_4_ | 358.2007 | 685.80 | [M+H]+ |
| CHSSC17 | Leonubiastrin | CC(C)C1=CC2=C(C(=C1)O)C3(C=CC(=O)C(C3C(C2O)OC(=O)C)(C)C(=O)OC)C | C_23_H_28_O_7_ | 415.1718 | 133.92 | [M-H]- |
| CHSSC18 | Luvangetin | CC1(C=CC2=C(O1)C(=C3C(=C2)C=CC(=O)O3)OC)C | C_15_H_14_O_4_ | 281.0807 | 688.64 | [M+Na]+ |
| CHSSC19 | Moracin C | CC(=CCC1=C(C=C(C=C1O)C2=CC3=C(O2)C=C(C=C3)O)O)C | C_19_H_18_O_4_ | 311.1271 | 590.78 | [M+H]+ |
| CHSSC20 | Nobiletin | COC1=C(C=C(C=C1)C2=CC(=O)C3=C(O2)C(=C(C(=C3OC)OC)OC)OC)OC | C_21_H_22_O_8_ | 403.1381 | 620.19 | [M+H]+ |
| CHSSC21 | Nodakenin | CC(C)(C1CC2=C(O1)C=C3C(=C2)C=CC(=O)O3)OC4C(C(C(C(O4)CO)O)O)O | C_20_H_24_O_9_ | 407.1347 | 455.38 | [M-H]- |
| CHSSC22 | Obacunone | CC1(C2CC(=O)C3(C(C2(C=CC(=O)O1)C)CCC4(C35C(O5)C(=O)OC4C6=COC=C6)C)C)C | C_26_H_30_O_7_ | 455.2067 | 636.95 | [M+H]+ |
| CHSSC23 | Ochratoxin B | CC1CC2=C(C(=C(C=C2)C(=O)NC(CC3=CC=CC=C3)C(=O)O)O)C(=O)O1 | C_20_H_19_NO_6_ | 370.1284 | 564.67 | [M+H]+ |
| CHSSC24 | Odoriflavene | COC1=C(C(=C(C=C1)C2=CC3=C(C=C(C=C3)O)OC2)O)OC | C_17_H_16_O_5_ | 301.1069 | 653.13 | [M+H]+ |
| CHSSC25 | Oxyberberine | COC1=C(C2=C(C=C1)C=C3C4=CC5=C(C=C4CCN3C2=O)OCO5)OC | C_20_H_17_NO_5_ | 352.1178 | 589.46 | [M+H]+ |
| CHSSC26 | Quercetin | C1=CC(=C(C=C1C2=C(C(=O)C3=C(C=C(C=C3O2)O)O)O)O)O | C_15_H_10_O_7_ | 303.0496 | 461.04 | [M+H]+ |
| CHSSC27 | ,6-Di-tert-butylhydroquinone | C(C)(C)C1=CC(=CC(=C1O)C(C)(C)C)O | C_14_H_22_O_2_ | 223.1695 | 533.18 | [M+H]+ |
| CHSSC28 | 1,7-Dihydroxy-3-methoxy-2-prenylxanthone | CC(=CCC1=C(C=C2C(=C1O)C(=O)C3=C(O2)C=CC(=C3)O)OC)C | C_19_H_18_O_5_ | 327.1227 | 664.25 | [M+H]+ |
| CHSSC29 | 1-Chloro-8-(3-heptyloxiran-2-yl)octa-4,6-diyne-2,3-diol | CCCCCCCC1C(O1)CC#CC#CC(C(CCl)O)O | C_17_H_25_ClO_3_ | 313.1550 | 512.15 | [M+H]+ |
| CHSSC30 | 2-Ethyl-3-methoxypyrazine | CCC1=NC=CN=C1OC | C_7_H_10_N_2_O | 139.0867 | 119.14 | [M+H]+ |
| CHSSC31 | 4-Methoxyphenylacetic acid | COC1=CC=C(C=C1)CC(=O)O | C_9_H_10_O_3_ | 165.0556 | 176.50 | [M-H]- |
| CHSSC32 | 4-tert-Butylphenyl salicylate | CC(C)(C)C1=CC=C(C=C1)OC(=O)C2=CC=CC=C2O | C17H18O3 | 271.1331 | 607.40 | [M+H]+ |
| CHSSC33 | 6-(1,1-Dimethylallyl)-2-(1-hydroxy-1-methylethyl)-2,3-dihydro-7H-furo[3,2-G]chromen-7-one | CC(C)(C=C)C1=CC2=CC3=C(C=C2OC1=O)OC(C3)C(C)(C)O | C_19_H_22_O_4_ | 313.1445 | 724.10 | [M-H]- |
| CHSSC34 | Baicalin | C1=CC=C(C=C1)C2=CC(=O)C3=C(C(=C(C=C3O2)OC4C(C(C(C(O4)C(=O)O)O)O)O)O)O | C_21_H_18_O_11_ | 445.0777 | 461.99 | [M-H]- |
| CHSSC35 | Biotin | C1C2C(C(S1)CCCCC(=O)O)NC(=O)N2 | C_10_H_16_N_2_O_3_S | 245.0955 | 270.48 | [M+H]+ |
| CHSSC36 | Buddlejasaponin Ivb | CC1C(C(C(C(O1)OC2CCC3(C(C2(C)CO)CCC4(C3C=CC5=C6CC(CCC6(C(CC54C)O)CO)(C)C)C)C)OC7C(C(C(C(O7)CO)O)O)O)OC8C(C(C(C(O8)CO)O)O)O)O | C_48_H_78_O_18_ | 943.5255 | 553.24 | [M+H]+ |
| CHSSC37 | Colnelenic acid | CCC=CCC=CC=COC=CCCCCCCC(=O)O | C_18_H_28_O_3_ | 293.2111 | 544.08 | [M+H]+ |
| CHSSC38 | Crispolide | CC12CCC(C(=CCC3C(C1O)OC(=O)C3=C)C2)OO | C_15_H_20_O_5_ | 281.1387 | 298.94 | [M+H]+ |
| CHSSC39 | ent-16-Kauren-19-ol acetate | CC(=O)OCC1(CCCC2(C1CCC34C2CCC(C3)C(=C)C4)C)C | C_22_H_34_O_2_ | 331.2633 | 1012.11 | [M+H]+ |
| CHSSC40 | gamma-Glutamylphenylalanine | C1=CC=C(C=C1)CC(C(=O)O)NC(=O)CCC(C(=O)O)N | C_14_H_18_N_2_O_5_ | 293.1239 | 237.45 | [M-H]- |
| CHSSC41 | Gibberellin A4&A7 | CC12C(C=CC3(C1C(C45C3CCC(C4)C(=C)C5)C(=O)O)OC2=O)O | C_19_H_22_O_5_ | 329.1390 | 544.64 | [M-H]- |
| CHSSC42 | Hesperetin | COC1=C(C=C(C=C1)C2CC(=O)C3=C(C=C(C=C3O2)O)O)O | C_16_H_14_O_6_ | 301.0716 | 394.60 | [M-H]- |
| CHSSC43 | Honyudisin | CC(=CCC1=C2C(=C3C(=C1O)C=CC(=O)O3)C=CC(O2)(C)C)C | C_19_H_20_O_4_ | 313.1433 | 544.52 | [M+H]+ |
| CHSSC44 | Icaritin | CC(=CCC1=C2C(=C(C=C1O)O)C(=O)C(=C(O2)C3=CC=C(C=C3)OC)O)C | C_21_H_20_O_6_ | 367.1290 | 658.55 | [M-H]- |
| CHSSC45 | Liquiritigenin | C1C(OC2=C(C1=O)C=CC(=C2)O)C3=CC=C(C=C3)O | C_15_H_12_O_4_ | 257.0807 | 356.16 | [M+H]+ |
| CHSSC46 | Nuciferine | CN1CCC2=CC(=C(C3=C2C1CC4=CC=CC=C43)OC)OC | C_19_H_21_NO_2_ | 296.1660 | 244.88 | [M+H]+ |
| CHSSC47 | Pelargonidin 3-glucoside ion | C1=CC(=CC=C1C2=[O+]C3=CC(=CC(=C3C=C2OC4C(C(C(C(O4)CO)O)O)O)O)O)O | C_21_H_21_O_10_ | 431.0986 | 357.12 | [M-2H]- |
| CHSSC48 | Physovenine | CC12CCOC1N(C3=C2C=C(C=C3)OC(=O)NC)C | C_14_H_18_N_2_O_3_ | 263.1389 | 196.45 | [M+H]+ |
| CHSSC49 | Pseudobaptigenin | C1OC2=C(O1)C=C(C=C2)C3=COC4=C(C3=O)C=CC(=C4)O | C_16_H_10_O_5_ | 281.0454 | 559.63 | [M-H]- |
| CHSSC50 | Valproic acid | CCCC(CCC)C(=O)O | C_8_H_16_O_2_ | 143.1076 | 390.40 | [M-H]- |
